# Reversible dendrite-free Li-plating/stripping electrochemistry achieved by stress-regulating carbon aerogel

**DOI:** 10.1093/nsr/nwaf305

**Published:** 2025-07-30

**Authors:** Lan-Xing Li, Ying-Xian Li, Yu-Shuai Feng, Ya-Hao Du, Peng Ouyang, Qin Chen, Hui Yang, Huan Ye, Fei-Fei Cao

**Affiliations:** College of Chemistry, Huazhong Agricultural University, Wuhan 430070, China; College of Chemistry, Huazhong Agricultural University, Wuhan 430070, China; College of Chemistry, Huazhong Agricultural University, Wuhan 430070, China; College of Chemistry, Huazhong Agricultural University, Wuhan 430070, China; State Key Laboratory of Material Processing and Die & Mould Technology, Department of Mechanics, School of Aerospace Engineering, Huazhong University of Science and Technology, Wuhan 430074, China; State Key Laboratory of Material Processing and Die & Mould Technology, Department of Mechanics, School of Aerospace Engineering, Huazhong University of Science and Technology, Wuhan 430074, China; State Key Laboratory of Material Processing and Die & Mould Technology, Department of Mechanics, School of Aerospace Engineering, Huazhong University of Science and Technology, Wuhan 430074, China; College of Chemistry, Huazhong Agricultural University, Wuhan 430070, China; College of Chemistry, Huazhong Agricultural University, Wuhan 430070, China

**Keywords:** lithium metal battery, lithium metal anode, dendrite suppression, carbon aerogel, kinetics

## Abstract

The stress distribution in Li metal strongly affects the interfacial Li-ion diffusion, thereby influencing the morphology of plated Li and the performance of the battery. Here, we report a mechano-electrochemical coupling strategy that utilizes an arched structured carbon aerogel to achieve stable Li-plating/stripping electrochemistry. The arch-structured carbon aerogel can actively regulate stress distributions in response to the compressive stresses induced by Li deposition, generating the transition of stress from compressive on the convex surface to tensile on the concave surface, which can effectively promote the Li-migration kinetics and thus suppress the non-uniform deposition of Li. The carbon aerogel with synergistically enriched-oxygen vacancies on its surface boosts rapid interfacial Li-ion migration by reducing the Li^+^ migration barrier. The non-dendritic Li-metal anode demonstrates smaller electrode level volume variation (<3%), higher coulombic efficiency (98.5%) and a longer cycle lifetime (2000 h at 1 mA cm^−2^) than conventional planar substrates. A full cell based on the LiFePO_4_ cathode shows a high capacity retention of 90.2% after 300 cycles at 1 C. The carbon-aerogel/Li|sulfurized polyacrylonitrile full cell delivers a reversible capacity of 1130 mA h g^−1^ over 270 cycles at 0.2 C. This work reveals a stress-driven dendrite growth suppression mechanism and provides insights into the design of dendrite-free metal anodes for rechargeable metal batteries.

## Teaser

An arch-structured carbon aerogel with enriched-oxygen vacancies is designed to relieve Li-plating-induced internal stress in Li metal and to achieve dendrite-free Li deposition.

## INTRODUCTION

Lithium-ion batteries are widely used to power portable electronics, but they are unsuitable energy-storage devices for longer standby times or driving ranges due to their limited energy density [1,2]. The low energy density is a result of the intercalation reactions occurring at the electrodes, which are responsible for energy storage and conversion. Li-metal batteries display a higher energy density via a conversion reaction mechanism, but their short cycling life and poor safety make them difficult to use for electric vehicles and grid storage [3–6]. Li-dendrite formation at the Li anode presents the most severe challenge, as Li dendrites cause internal short circuits between the Li anode and cathode [7,8]. They also pose severe safety hazards and give rise to serious capacity fading by consuming both active Li and the electrolyte [9–11]. Continuous plating and stripping of Li dendrites produce a porous Li electrode and result in an infinite relative volume change of the Li anodes, aggravating Li-anode pulverization and causing rapid battery performance degradation [12].

**Figure 1.**
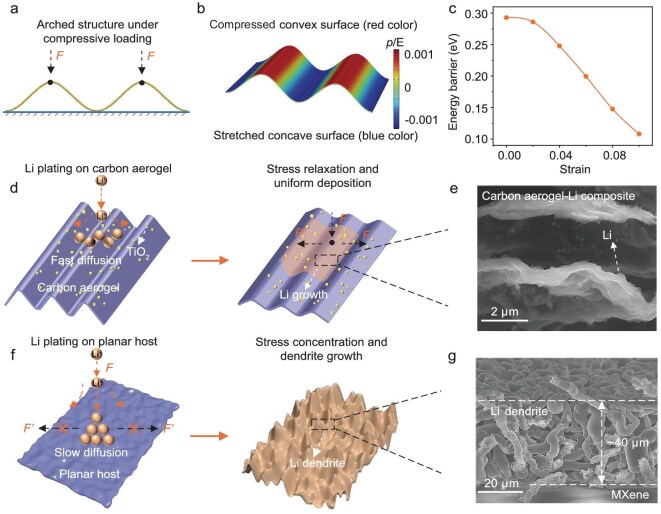
Li-plating behavior on two substrates. (a) Schematic diagram of stress analysis of an arched structure under compressive loading. (b) Finite element simulation for pressure distribution of the arched structure subjected to compressive loading, showing the transition of the stress/strain from compressive on the convex surface to tensile on the concave surface. The pressure is normalized by using the Young's modulus of the material. (c) Evolution of Li-migration energy barrier on the surface of arch-structured carbon aerogels under tensile stress/strain. Schematic diagram of Li plated on (d) a substrate with a wave-shaped layered structure and (f) a substrate with a parallel layered structure. Li exhibits dendritic morphology on the planar host due to localized stress concentration, whereas uniform Li deposition occurs on carbon aerogel as a result of stress regulation and relaxation. (e) Side-view scanning electron microscope (SEM) image of uniform Li deposits of 6 mA h cm^−2^ on a substrate with a wave-shaped layered structure. (g) Side-view SEM image of uniform 6 mA h cm^−2^ of dendritic Li deposited on a substrate with a tightly parallel layered structure. The current density is 0.5 mA cm^−2^.

Li prefers to grow into a dendritic morphology driven by thermodynamic factors such as a weak interaction energy and high diffusion barrier [13,14]. 3D structural Li anodes have been rationally designed to address this issue, but most studies have focused on controlling the homogeneous nucleation and early-stage growth of Li [15–18]. Less attention has been paid to the plating-induced stress in Li metal and this stress is strongly correlated with the Li-deposition morphology [19,20]. Defects or protuberances on Li substrates can induce Li-dendrite growth from their bases during electrochemical deposition and such non-uniform Li deposition creates areas with large internal compressive stress that can initiate cracks in the Li anodes. There may also be large internal stresses due to hostless Li plating/stripping, which produces huge electrode volume changes [21,22]. The transport of lithium atoms is hindered by the accumulated stress and Li grows along the fractures in the form of dendrites, degrading the performance of the battery and posing safety hazards. To increase the cyclic stability and safety of Li-metal anodes, carbon-based materials that can release stress have been applied to alleviate Li-dendrite formation and growth [23–25]. However, due to their high stiffness and poor lithiophilicity, most carbon-based materials are unsuitable for use as hosts for Li metal. In contrast, elastic and lithiophilic carbon aerogels that can relieve accumulated stress during continuous deposition show great promise for use as Li-anode hosts. As carbon aerogels with diverse microstructures may exhibit distinct mechanical properties, we investigate the stress distribution in an arched structure by using finite element analysis. The calculation results reveal that, when the surface of the arched structure is subjected to compressive loading (Fig. 1a) when in contact with the counter electrode through a separator, the structure easily undergoes geometric deformation, not only effectively mitigating the stress concentration at the contact points, but also generating the transition of stress/strain from compressive on the convex surface to tensile on the concave surface (Fig. 1b). The presence of tensile stress/strain significantly reduces the migration energy barrier of Li atoms and the extent of this reduction becomes more pronounced with increasing stress/strain (Fig. 1c). Therefore, the arched structure with regulated stress gradient and tensile stress can facilitate the rapid migration of Li atoms, ultimately leading to the uniform deposition of Li, in comparison with dendritic Li for the planar host (Fig. 1d and e). In contrast, when a planar structure is subjected to the same loading (Fig. S1a), the produced compressive stress tends to be concentrated predominantly in the local region, without a large-scale stress gradient generated (Fig. S1b). Such localized stress accelerates preferential lithium growth around the loaded local region and, in turn, the localized lithium deposition can aggravate the stress concentration, finally leading to the formation of dendritic morphologies (Fig. 1f and g).

Inspired by the above analysis, we herein report an arch-structured carbon aerogel for use as a Li-metal anode to release accumulated stresses and ensure uniform Li plating. The carbon aerogel is composed of staggered layered structures that deform and dissipate energy after compressive stress during continuous Li deposition. This prevents Li-dendrite deposition caused by the localized stress concentration. Lithiophilic TiO_2_ nanoparticles with enriched-oxygen vacancies are highly dispersed on the staggered layers of the carbon aerogel, which promotes the preferential adsorption and rapid migration of Li atoms on the carbon aerogel during the nucleation stage. This helps guide subsequent non-dendritic Li deposition during the growth stage. Moreover, this layered carbon aerogel offers elastic and stable skeletons for Li plating and stripping, thus mitigating a huge volume change in the Li anode. As a result, the Li–carbon aerogel composite anode exhibits only a 3% thickness fluctuation, even after being plated with a high areal capacity of Li of 6 mA h cm^−2^ (Fig. S2). The Li|Li symmetric cells show a lifetime of >2000 h at a current density of 1 mA cm^−2^ with an aerial capacity of 1 mA h cm^−2^. In addition, a Li–carbon aerogel|LiFePO_4_ full cell demonstrates a high capacity retention of 90.2% for 300 cycles at 1 C.

## RESULTS AND DISCUSSION

### Structural analyses

**Figure 2.**
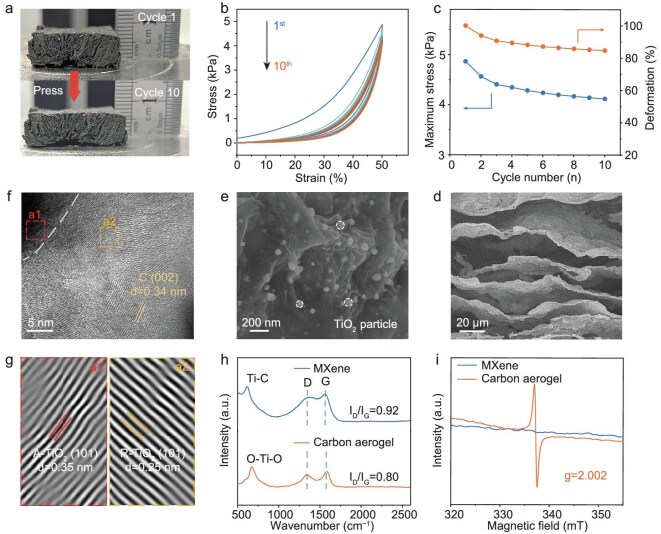
Structural characterization of carbon aerogel. (a) Optical image of the compression and elasticity behavior of carbon aerogel. (b) Stress–strain curves of carbon aerogel for 10 cycles. (c) Stress retentions of carbon aerogel after compression for 10 cycles at a strain of 50%. SEM image of carbon aerogel at (d) low magnification and (e) high magnification. (f, g) High-resolution transmission electron microscopy images of carbon aerogel. (h) Raman spectra of MXene and carbon aerogel. (i) EPR spectra of MXene and carbon aerogel.

A compressible elastic carbon aerogel was synthesized by using a mixing–casting method, directional freezing and final pyrolysis, as illustrated in Fig. 2a and Fig. S3. The negatively charged Ti_3_C_2_T*_x_* (MXene) interacts with chitosan via electrostatic interactions to improve the mechanical strength of the carbon aerogel [26]. Cyclic mechanical test results, as shown in Fig. 2b and c, indicate that the carbon aerogel could withstand 10 compression–release cycles at a compression strain of 50% and retain 86% of its initial stress. A hysteresis loop related to typical viscoelastic behavior is observed for the carbon aerogel, suggesting that stress relaxation occurs [27]. The carbon aerogel shows good structural integrity, even after being repeatedly bent to various angles, showing its promising potential as a host to stabilize composite Li-metal anodes (Fig. S4).

The carbon aerogel shows a 2D structure composed of staggered wave-shape layers (Fig. 2d). The staggered wave-shape layers are several microns in diameter and have TiO_2_ nanoparticles with an average size of 50 nm well dispersed on the surface (Fig. 2e). This can be verified by the uniform distribution of C, O and Ti elements in the energy-dispersive spectroscopy element maps (Fig. S5). The high-resolution transmission electron microscopy images, as shown in Fig. 2f and g, show two sets of parallel fringes with an interplanar distance of 0.25 and 0.35 nm. These distances correspond to the (101) facet of rutile-TiO_2_ (R-TiO_2_) (JCPDS 21–1276) and anatase-TiO_2_ (A-TiO_2_) (JCPDS 21–1272) in the X-ray diffraction patterns (Fig. S6), respectively [28]. Fourier transform analysis indicates that both R-TiO_2_ and A-TiO_2_ exhibit lattice distortions and twisting, indicating the presence of defects in the TiO_2_ (Fig. 2g).

The chemical composition and surface defects in the TiO_2_ of the carbon aerogel were investigated by using Raman spectroscopy, electron paramagnetic resonance spectroscopy (EPR) and X-ray photoelectron spectroscopy (XPS). The compacted MXene host prepared by using vacuum filtration was used for comparison (Fig. S7). In the Raman spectra (Fig. 2h), the vibrational peak at 622.8 cm^–1^ corresponds to the Ti–C bond of the MXene [29]. For the carbon aerogel anchored to TiO_2_ nanoparticles, the peak at 675.5 cm^–1^ is attributed to the symmetric stretching and bending vibrations of the O–Ti–O bonds in A-TiO_2_. No vibrational peak of Ti–C bonds is observed, indicating the complete conversion of the MXene to the carbon aerogel. Additionally, the conversion of the MXene to a carbon aerogel improves the intrinsic electronic conductivity of the material, as illustrated by a lower intensity ratio (*I*_D_/*I*_G_) of the D and G peaks. The higher electronic conductivity is due to the existence of unpaired electrons in the carbon aerogel. The carbon aerogel shows a representative EPR signal at a *g*-factor of 2.002 (Fig. 2i), indicating the existence of unpaired electrons in the TiO_2_, which are oxygen vacancies (OVs) [30]. In contrast, the MXene host demonstrates no evidence of OVs (Fig. 2i). XPS spectra were conducted to investigate the OVs in the TiO_2_ of the carbon aerogel. A peak at 456 eV in the Ti 2p core-level XPS spectra and another at 532.2 eV in the O 1s core-level XPS spectra of the carbon aerogel are related to the presence of OVs (Fig. S8) [31]. The OV concentration in the TiO_2_ of the carbon aerogel reaches 22%. A high OV concentration facilitates rapid charge transfer in the carbon aerogel and improves the Li-plating/stripping kinetics [32]. The carbon aerogel maintains a moderate specific surface area (107.2 m^2^ g⁻¹) while exhibiting a high porosity of 94.5% (Fig. S9). This optimal balance between the surface area and the porosity not only mitigates excessive solid–electrolyte interphase (SEI) formation at the carbon host–electrolyte interface, but also provides sufficient space to accommodate high areal capacities of Li.

### Li-metal deposition electrochemistry

**Figure 3.**
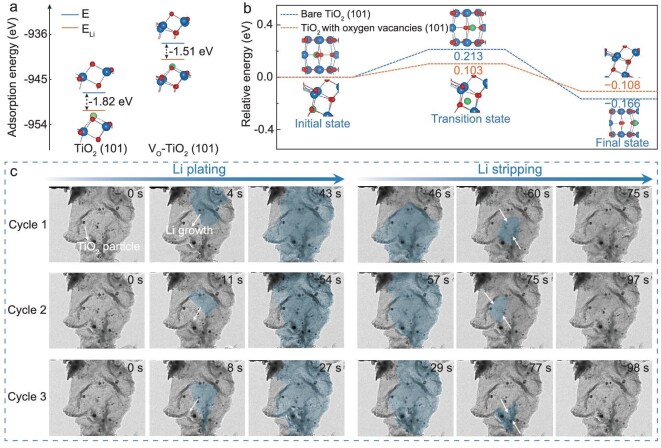
(a) Calculated adsorption energies of Li on TiO_2_ with (V_o_–TiO_2_) and without (TiO_2_) OVs. The green, red and blue balls represent lithium, oxygen and titanium atoms, respectively. (b) Li-migration energy pathways and barriers on V_o_–TiO_2_ and TiO_2_. (c) *In situ* high-resolution transmission electron microscopy images of Li-metal plating and stripping along the 2D wave-shape layers of carbon aerogel during the initial three cycles. The deposited Li is colored with blue for clear observation and the plating/stripping pathways are marked by white arrows.

The host architecture greatly affects Li nucleation and growth, and can impact the final Li-plating morphology. For comparison, an MXene that was not compressible or elastic was used as a reference. The carbon aerogel is both highly compressible and elastic, and produces horizontal tensile stress on Li atoms and prevents their aggregation on the substrate [33]. This results in the uniform nucleation of Li atoms on the substrate, followed by the non-dendritic growth of Li. In contrast, Li grows into dendrites on the planar MXene due to the high diffusion energy barrier of the Li atoms. We carried out density functional theory (DFT) calculations to investigate the effect of OVs of the carbon aerogel on Li-atom diffusion and found that it has a low binding energy of −1.51 eV compared with the OV-free host (−1.82 eV) (Fig. 3a). Both binding energy values are negative, suggesting that the adsorption of Li atoms is thermodynamically favorable. The diminished adsorption interaction between the carbon aerogel and the Li indicates faster diffusion of the Li, resulting in a lower migration barrier of 0.103 eV in contrast to 0.213 eV for the OV-free host (Fig. 3b). The combination of lower binding energy and a reduced migration barrier for Li atoms on the carbon aerogel facilitates the desorption and diffusion of Li atoms from the substrate, thus ensuring uniform Li deposition [34].

*Ex situ* scanning electron microscopy was conducted to characterize the Li morphology on a carbon-aerogel sample with a stress relaxation function and an MXene sample that could not undergo stress relaxation. As the deposition capacity increased from 2 to 10 mA h cm^−2^ at a current density of 0.5 mA cm^−2^, the Li metal was uniformly deposited with a non-dendritic morphology on the 2D wave-shaped layers (Fig. S10). Even at high current densities of 2 and 4 mA cm^−2^, uniform Li deposition can still be achieved, demonstrating that the elastic carbon aerogel effectively facilitates rapid lithium-ion transfer at high rates (Fig. S11). This compact deposition also ensures a relatively stable electrode volume, with only a 3% thickness fluctuation even after plating 6 mA h cm^−2^ of Li. For the ester carbonate electrolyte case, non-dendritic Li is uniformly distributed on the staggered wave-shape layers of the carbon aerogel (Fig. S12). This shows the advantage of the carbon aerogel for ensuring dimensional stabilization (Fig. S13). In contrast, numerous Li filaments with a length of ≤50–100 μm are formed on the MXene (Fig. S14) because the slow transport kinetics enables localized Li deposition on the MXene surface. Dendritic Li protrudes from the MXene surface and grows into a porous structure, leading to a 444% thickness fluctuation.

To visualize the Li-deposition evolution, an electrochemical dry cell using the carbon aerogel attached to a Mo grid as the working electrode and Li metal decorated with a surface Li_2_O layer as the counter electrode was fabricated for *in situ* TEM characterization (Fig. 3c). Before the Li/Li_2_O electrode was moved into contact with the carbon aerogel, no Li deposits were observed on the carbon layers (0 s for cycling). When a bias voltage of 3 V was applied to the carbon aerogel, Li started to nucleate on the wrinkles and TiO_2_ nanoparticles of the 2D wave-shaped layers due to their low nucleation barrier (Fig. 3c and Supplementary Video S1). Then, the Li metal grows laterally up and gradually covers the carbon layers, prolonging the plating time (as highlighted by the blue region in Fig. 3c). The deposited Li does not grow into typical whisker shapes on the wrinkles because the 2D wave-shaped layers could deform and produce horizontal tensile stress on the Li atoms after undergoing deposition-induced compressive stress. During the stripping process, Li deposited on the carbon layers is reversibly stripped without leaving behind any Li residue. The 2D wave-shaped layers maintain their layered structure while the V_o_–TiO_2_ nucleation sites remain in contact with the carbon layers (Fig. S15), indicating that Li could be reversibly plated/stripped during long-term cycling. In the next two cycles on the same 2D wave-shaped carbon-aerogel layers, Li displays the same plating and stripping behavior due to the synergistic action of the carbon layers and V_o_–TiO_2_ nucleation sites (Fig. 3c and Videos S2 and S3). The Li metal remains in strong contact with the 2D wave-shaped carbon-aerogel layers throughout continuous cycling, indicating considerable affinity between the Li and the carbon substrate, as verified by the DFT calculations presented in Fig. 3a.

**Figure 4.**
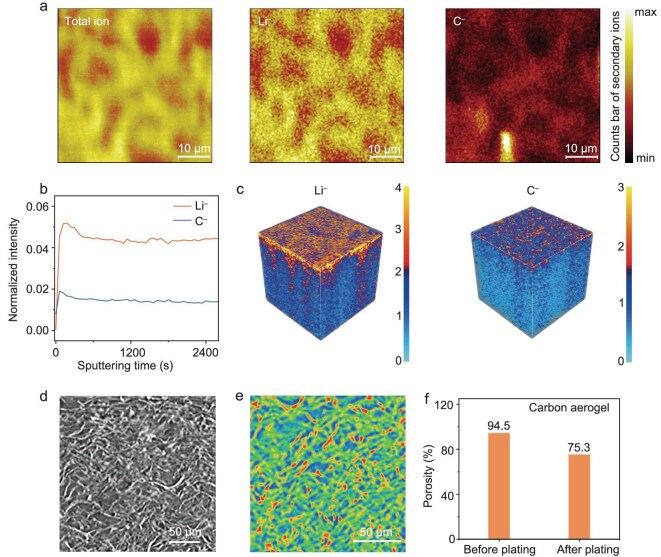
(a) Mapping distributions of the carbon-aerogel/Li surface were obtained by using time-of-flight secondary ion mass spectrometry (TOF-SIMS). (b) Depth profiles of TOF-SIMS sputtering on the surface of the carbon-aerogel/Li. (c) 3D reconstruction of TOF-SIMS corresponding to the depth profile in (b), where the size of the 3D model was *X* = 50 μm, *Y* = 50 μm, *Z* ∼ 3.23 μm. (d) Morphology of carbon aerogel after deposition of 6 mA h cm^−2^ of Li by X-ray microscopy and (e) corresponding color rendering. (f) Porosity of carbon aerogel before and after deposition of 6 mA h cm^−2^ of Li.

To elucidate the spatial distribution of the Li-metal deposition within the carbon-aerogel host, time-of-flight secondary ion mass spectrometry was employed to map the elemental distribution of C and Li in the composite Li-metal anode (6 mA h cm^−2^) integrated with the carbon-aerogel current collector. The C^−^-ion image reveals the 3D carbon framework and its interconnected porous architecture, while the Li distribution indicates preferential plating onto the carbon skeleton, effectively filling the pores of the carbon-aerogel matrix (Fig. 4a). Depth profiling analysis of the carbon-aerogel/Li composite anode demonstrates that the intensity trends of the C^−^ and Li^−^ ions exhibit strong correlation throughout the sputtering process, confirming the homogeneous infiltration and uniform distribution of Li within the carbon-aerogel scaffold (Fig. 4b and c). Furthermore, the structural evolution of the Li-deposited carbon aerogel was quantitatively characterized by using non-destructive X-ray microscopy (Fig. 4d and e). Comparative analysis reveals that the carbon-aerogel/Li composite anode maintains a thickness that is comparable to that of the pristine electrode, while exhibiting a significant reduction in porosity after Li plating, as evidenced by the decreased void fraction (Fig. 4f).

### Kinetics

**Figure 5.**
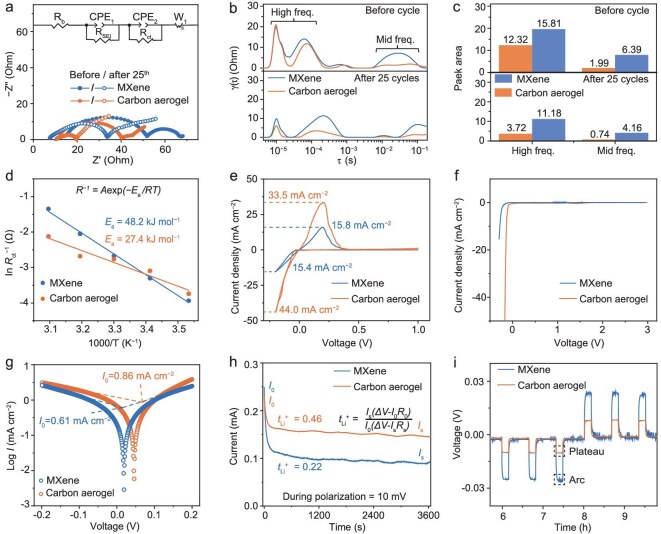
(a) Nyquist impedance plots of carbon aerogel and MXene-based Li|Li symmetric cells before and after 25 cycles at 1 mA cm^−2^ with a Li-plating capacity of 1 mA h cm^−2^. (b) DRT analysis fitted from EIS plots as shown in Fig. 5a. (c) Peak area comparison of the four peaks within a timescale (*τ*) range of 10^−5^–10^−1^ s based on DRT analysis. (d) Activation energy comparison for the carbon-aerogel- and MXene-based Li|Li symmetric cells. (e) Cycle voltammetry of carbon-aerogel|Li and MXene|Li half cells at a scan rate of 0.2 mV s^−1^. (f) Linear sweep voltammetry of carbon-aerogel|Li and MXene|Li half cells between 3.0 and −0.3 V at a scan rate of 0.2 mV s^−1^. (g) Tafel curves for carbon-aerogel- and MXene-based Li|Li symmetric cells. (h) Chronoamperometry results to compare Li^+^-transference numbers (t_Li_^+^) at 10 mV of polarization. (i) GITT plots of carbon-aerogel- and MXene-based Li|Li symmetric cells at 1 mA cm^−2^ and 1 mA h cm^−2^. The cells were periodically plated and stripped every 10 min followed by a 30-min rest.

Parallel microchannels in the carbon aerogel improve the electrolyte wettability (Fig. S16) and enable enhanced Li^+^-transfer kinetics. The Li^+^-transfer kinetics of the carbon aerogel were scrutinized by using electrochemical impedance spectroscopy (EIS) for the Li|Li symmetrical cells. The carbon aerogel exhibits lower initial charge-transfer resistance (*R*_ct_) than the MXene. Even after 25 cycles, the *R*_ct_ of the carbon aerogel remains much lower than that of the MXene (Fig. 5a and Table S1), implying faster Li-plating/striping kinetics on the carbon aerogel. The distribution of relaxation time (DRT) fitted from the EIS spectra was conducted to decouple the electrochemical processes (Fig. 5b). The DRT plots of the carbon aerogel and the MXene before and after 25 cycles display four peaks within a timescale (*τ*) range of 10^−5^–10^−1^ s. The *τ*1 peak at 10^−5^ s is ascribed to the contact resistance, the *τ*2 peak at 10^−4^–10^−3^ s is assigned to the Li^+^ transport across the solid–electrolyte interphase (SEI) layer, and the *τ*3 and *τ*4 peaks at 10^−2^–10^−1^ s are related to the *R*_ct_ [35,36]. The four peaks decrease after cycling, demonstrating better solid–solid contact, decreased SEI-layer resistance and enhanced charge-transfer kinetics. The four peaks of the carbon aerogel are much lower than that of the MXene (Fig. 5c), suggesting the better electrochemical performance of the carbon aerogel [37]. The composition of the SEI was investigated through compositional analysis performed via XPS. The XPS spectra of the surface layers indicate that the SEI for both substrates, formed from the reaction between the ether-based electrolyte and the Li metal, is predominantly composed of ROCO_2_Li, −CF_3_, Li_2_CO_3_ and LiF (Fig. S17). The SEI generated from the carbon aerogels contains a balanced mixture of organic (C–O, C=O) and inorganic (LiF, Li_2_CO_3_) components, exhibiting significantly higher contents than those found in the MXene. The presence of inorganic components is advantageous for suppressing Li-dendrite formation, while organic constituents enhance mechanical flexibility, allowing the structure to accommodate volume variations and mechanical stress [38,39]. This hybrid SEI architecture synergistically stabilizes the electrode–electrolyte interface, alleviates dendrite formation and reduces side reactions, thereby highlighting the superiority of the carbon aerogels over the MXene in LMBs.

Temperature-dependent EIS was carried out to investigate the Li^+^-transfer kinetics. The activation energy (*E*_a_) determined from fitting the EIS spectra was used to evaluate Li^+^ transport across the SEI. The carbon aerogel shows an *E*_a_ of 27.4 kJ mol^−1^, which is much lower than that of the MXene (48.2 kJ mol^−1^) (Fig. 5d), indicating faster Li^+^-diffusion kinetics in the carbon aerogel [40]. We used cyclic voltammetry (CV) to investigate the Li^+^-transfer kinetics and reaction efficiency of the carbon aerogel. As expected, the carbon-aerogel|Li half cells show a much higher current density compared with the MXene|Li half cells, indicating faster Li-plating/stripping electrochemistry (Fig. 5e). Additionally, the peak area of the anodic CV for the carbon-aerogel|Li half cells is larger than that of the MXene|Li half cells, demonstrating that the carbon aerogel facilitates better reversibility of the plated Li. In addition, the CV curves of the carbon-aerogel|Li and MXene|Li half cells at different sweep rates (Fig. S18a and b) were collected for comparison. Compared with the cells when using the carbon aerogel, the MXene exhibits a larger shift in the redox peaks and more severe capacity degradation at higher sweep rates. As shown in Fig. S18c and d, a larger slope (*b*-value) of the fitted line indicates a higher diffusion coefficient. The fitted *b*-value of the carbon aerogel is higher than that of the MXene, which suggests that the diffusion kinetics of the carbon-aerogel-based battery are improved, with sufficient Li^+^ near the lithium surface during the deposition/stripping process. The kinetics of the Li plating/stripping were also analysed by using linear sweep voltammetry. The carbon-aerogel|Li half cells display a lower overpotential and higher response current than the MXene|Li half cells (Fig. 5f). This difference indicates a reduced nucleation and growth barrier, as well as enhanced diffusion kinetics [41]. The exchange current densities (*I*_0_) of the carbon aerogel and the MXene were obtained by fitting the Tafel plots, and the carbon aerogel reaches *I*_0_ = 0.86 mA cm^−2^. This value surpasses that of the MXene (0.61 mA cm^−2^) (Fig. 5g), indicating faster Li^+^-diffusion and Li-plating/stripping kinetics in the carbon aerogel [42].

The Li^+^-transference number (t_Li_^+^) of the carbon aerogel is t_Li_^+^ = 0.46, which is better than that of the MXene (0.22), indicating faster Li^+^ migration in the carbon aerogel (Fig. 5h) [43]. We also conducted a galvanostatic intermittent titration technique (GITT) to compare the Li-plating/stripping kinetics in the two Li|Li symmetric cells (Fig. 5i and Fig. S19). The GITT profiles of the MXene-based Li|Li symmetric cells exhibit higher voltage polarization compared with those of the carbon-aerogel-based Li|Li symmetric cells, which is primarily due to sluggish Li^+^ transport across the MXene electrode. The accumulation of inactive Li is responsible for mass transport limitations during cycling. Li preferentially plates as long, filament-like dendrites on the MXene electrode (Fig. S14), which can become mechanically detached from the electrode surface, forming electrically isolated Li. The formation of a tortuous dead Li layer blocks Li^+^ transport and leads to an arcing voltage profile as cycling progresses. In contrast, the carbon-aerogel-based Li|Li symmetric cells show no peaking behavior associated with dendrite formation, demonstrating that mass and charge transfers across the carbon-aerogel electrode occurs homogeneously. The overpotential and ohmic polarization of the carbon aerogel are much lower than those of the MXene (Fig. S20), indicating smaller charge-transfer resistance and contact resistance of the carbon aerogel [44]. This is in agreement with the EIS results, verifying the superior Li^+^-diffusion kinetics of the carbon aerogel.

### Cell performances

**Figure 6.**
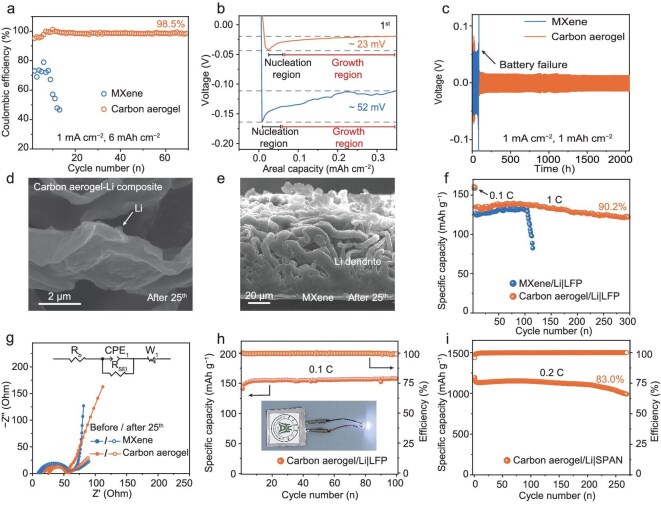
(a) Coulombic efficiencies of carbon aerogel and MXene at 1 mA cm^−2^ with a Li-plating capacity of 6 mA h cm^−2^. (b) Corresponding voltage profiles of carbon aerogel and MXene. (c) Comparison of voltage profiles of carbon-aerogel- and MXene-based Li|Li symmetric cells with a Li-plating capacity of 1 mA h cm^−2^ at 1 mA cm^−2^. SEM images of (d) carbon-aerogel and (e) MXene electrodes after 25 cycles. The current density is 1 mA cm^−2^ and the areal capacity is 1 mA h cm^−2^. (f) Long-term cycling performance of full cells for carbon aerogel and MXene anodes by using LiFePO_4_ as the cathode at 1 C in the voltage range of 2.0−4.0 V. (g) Nyquist impedance plots of full cells for carbon-aerogel and MXene anodes before and after 25 cycles at 1 C. (h) Cycle performance of the carbon-aerogel/Li|LFP pouch cell at 0.1 C. (i) Cycle performance of the carbon-aerogel/Li|SPAN full cell at 0.2 C.

Due to the faster Li^+^-transfer kinetics and the absence of Li dendrites on the elastic carbon-aerogel substrate, improved cycling performances in terms of lower nucleation overpotential and higher coulombic efficiency (CE) on the carbon aerogel are achieved (Fig. 6a and b). The carbon aerogel can deliver a high specific gravimetric capacity of ∼1920 mAh g^−1^ based on the whole composite anode. This composite capacity significantly exceeds those of other composite lithium anodes, including 549 mAh g^−1^ for Li/Cu, 1088 mAh g^−1^ for Li/carbon aerogel without TiO_2_, 1703 mAh g^−1^ for Li/copper nanowire-phosphide, 1580 mAh g^−1^ for Li/MXene/silver nanowire, 1736 mAh g^−1^ for Li/carbon nanofiber and 913 mAh g^−1^ for Li/graphite microtubes (Fig. S21) [45–48]. At a current density of 1 mA cm^−2^ and an aerial capacity of 6 mA h cm^−2^, the carbon aerogel shows a stable CE of 98.5% for 70 cycles. In contrast, the MXene rapidly fails after only eight cycles. The planar Cu electrodes exhibit rapid performance degradation, with the Li-plating/stripping efficiency decreasing from 90% to merely 72% within 20 cycles. This deterioration can be attributed to the inherent instability of both the dendritic Li morphology and the dynamic anode/electrolyte interface during repeated cycling (Fig. S22). To highlight the superior performance of the carbon aerogel integrated with oxygen-deficient TiO_2_ (V_o_–TiO_2_), comparative studies were conducted by using a homemade carbon aerogel without V_o_–TiO_2_ and commercial carbon-aerogel powders without V_o_–TiO_2_. The nucleation overpotential of the carbon aerogel with V_o_–TiO_2_ is significantly lower than that of the V_o_–TiO_2_-free counterparts, underscoring the critical role of V_o_–TiO_2_ in facilitating efficient and uniform Li nucleation (Fig. S23 and Fig. 6b). For higher areal capacities of 18 mA h cm^−2^, the carbon-aerogel electrode still shows stable cycling with a CE of 98.3%, while only 12 mA h cm^−2^ is obtained when using the MXene electrode (Fig. S24).

The electrochemical performances of the carbon aerogel and the MXene were further compared by using symmetric cells. When 1 mA h cm^−2^ of Li was reversibly plated and stripped between the carbon aerogel at 1 mA cm^−2^, the carbon aerogel stably cycled for ≤2000 h (related to the stable voltage profiles), which is nearly 10 times that of the MXene (<200 h) (Fig. 6c). As the plating capacity is further increased to 6 mA h cm^−2^, cycling for >750 h and a low overpotential of <40 mV are achieved (Figs S25–S27), which is superior to those of the MXene electrode. The stable cycling of the carbon-aerogel electrode arises from the homogeneous Li deposition (Fig. 6d), affording the electrode with less Li and electrolyte depletion. The fast decay in battery performances for the MXene electrode is due to the growth of Li dendrites and the consumption of the electrolyte caused by the reaction between dendrites and the electrolyte (Fig. 6e and Fig. S28).

To investigate the application feasibility of the carbon aerogel, full cells were assembled with carbon-aerogel/Li or MXene/Li anodes (6 mA h cm^−2^ of Li metal was pre-deposited on the substrates) and high loading of LiFePO_4_ (LFP) cathodes (>2 mA h cm^−2^). The full-cell performance of the carbon-aerogel/Li anode is superior to that of MXene, including a high capacity retention of 87.7% after 300 cycles at 0.5 C (Fig. S29). In comparison, the MXene/Li battery exhibits rapid capacity decay after the 180th cycle. The performance difference is further magnified by increasing the current rate to 1 C, where the cell with carbon-aerogel/Li anode delivers a reversible capacity of 145 mA h g^−1^, which is twice that of the MXene/Li anode (70 mA h g^−1^) (Fig. S30). The battery with a carbon-aerogel/Li anode stably cycles, showing 90.2% capacity retention after 300 cycles, while the MXene/Li|LFP full cell shows a much shorter life of ∼105 cycles (Fig. 6f). Batteries in which a carbon-aerogel/Li anode is utilized demonstrate long-term stability that surpasses that of previously reported batteries in other composite lithium anodes are employed (Table S2) [49–52]. The current density and capacity retention of the battery with a carbon-aerogel/Li anode exceeded those of reported Li-metal batteries with advanced composite Li anodes [50,53–55].

The realization of high-energy-density Li-metal batteries necessitates the achievement of both a low N/P ratio and lean-electrolyte conditions. To address this challenge, we constructed a Li-metal full-cell configuration exhibiting a 90% Li-utilization rate. The full cell incorporates a delithiated LiFePO₄ cathode (3.5 mA h cm^−2^, 23 mg cm^−2^) paired with a carbon-aerogel anode pre-deposited with 3.85 mA h cm^−2^ of Li. This configuration, featuring an N/P ratio of ∼0.1, delivers a high reversible capacity of 150 mA h g^−1^ at 0.1 C and maintains 80% of its initial capacity after 30 cycles (Fig. S31). To further evaluate the electrochemical performance under practical lean-electrolyte conditions, we systematically optimized the electrolyte parameters. While maintaining the areal loading of the LFP cathode at 15–16 mg cm^−2^, the electrolyte volume was significantly reduced from 45 to 25 μL per cell, corresponding to a decrease in the electrolyte-to-capacity ratio from 3.8 to 2.0 μL mg^−1^ (based on electrodes with a diameter of 10 mm). Notably, even under these stringent lean-electrolyte conditions, the carbon-aerogel/Li|LFP full cell demonstrates exceptional cycling stability, retaining a reversible capacity of 147.4 mA h g^−1^ after 50 cycles at 0.1 C with a capacity retention of 96% (Fig. S32) and 130 mAh g^−1^ after 110 cycles at 0.2 C with a high capacity retention of 90%. This remarkable performance under reduced electrolyte conditions not only highlights the superior electrolyte utilization efficiency enabled by the carbon-aerogel-anode architecture, but also underscores its significant potential for enabling practical high-energy-density Li-metal batteries. The ability to maintain stable cycling performance under such demanding conditions represents a critical step toward achieving the energy density target of 500 Wh kg^−1^.

We conducted EIS and compared the Li^+^-transfer kinetics of the two full cells before and after cycling. A lower *R*_ct_ was obtained for the carbon-aerogel/Li|LFP full cell compared with the MXene/Li|LFP full cell, implying faster charge transfer of Li^+^ in the carbon aerogel. The Nyquist impedance plots before and after cycling also confirm that no internal short-circuiting occurred during cycling (Fig. 6g and Table S3) [56]. These results indicate that the failure of the MXene battery is caused by the depletion of the electrolyte and Li dendrites (Fig. S33). The intact structure of the carbon aerogel and the homogeneous non-dendritic Li deposition endow the battery with superior rate capability (Fig. S34). The carbon-aerogel/Li|LFP full cell delivers a much higher capacity at a high rate (118 mA h g^−1^ at 2 C and 96 mA h g^−1^ at 3 C). In contrast, the MXene/Li|LFP full cell only provides 75 and 25 mA h g^−1^ at 2 and 3 C, respectively. Due to the merits of the carbon aerogel, we developed a foldable carbon-aerogel/Li|LFP pouch cell (Fig. S35) that delivers a reversible specific capacity of 155 mA h g^−1^ for 100 cycles at 0.1 C (Fig. 6h). Due to the lightweight advantage of the carbon aerogel compared with those of commercial current collectors including Al, Cu and porous carbons, an Ah-level carbon-aerogel/Li|LFP pouch cell is expected to deliver a significantly higher energy density (Fig. S36 and Table S4). To assess its practical feasibility, the carbon-aerogel/Li anode was paired with a sulfurized polyacrylonitrile (SPAN) cathode. As shown in Fig. 6i, the carbon-aerogel/Li|SPAN full cell achieves a super-high composite capacity of 1194 mA h g^−1^ and maintains a capacity of 1130 mA h g^−1^ over 270 cycles at 0.2 C, corresponding to a high capacity retention of 83%. Furthermore, the surface morphologies of the Li-metal anodes in the carbon-aerogel/Li|SPAN full cells were examined after cycling. The carbon-aerogel/Li anode exhibits a relatively flat and smooth surface, with no significant formation of lithium dendrites (Fig. S37).

## DISCUSSION

In summary, we have reported a carbon aerogel to relieve Li-plating-induced internal stress in Li metal and achieve dendrite-free Li deposition. The compressible and elastic carbon aerogel is composed of staggered wave-like layered structures, and deforms and generates horizontal tensile stresses on Li atoms once it is subjected to compressive stress. This prevents the aggregation of Li atoms on the substrate. The uniform distribution of Li atoms driven by the tensile stress leads to the uniform nucleation of Li atoms and the non-dendritic growth of Li on the carbon-aerogel substrate. As a result, a dendrite-free Li anode with long-term cycling of ≤2000 h and a small electrode volume change of <3% is achieved. Based on this anode configuration, the carbon-aerogel/Li|LFP full cell offers a high capacity retention of 90.2% for 300 cycles at 1 C. This work discloses a stress-driven Li-dendrite suppression mechanism and provides a feasible strategy for fabricating high-performance composite Li anodes.

## Supplementary Material

nwaf305_Supplemental_File
