# Supplementary material for: Reversible dendrite-free Li-plating/stripping electrochemistry achieved by stress-regulating carbon aerogel
Source: Natl Sci Rev. 2025 Jul 30;12(9):nwaf305. doi: 10.1093/nsr/nwaf305 (PMC12416278; doi:10.1093/nsr/nwaf305)
Supplement: nwaf305_Supplemental_File [file nwaf305_supplemental_file.pdf]

# Supporting Information

## Reversible Dendrite-Free Li Plating/Stripping Electrochemistry Achieved by Stress-Regulating Carbon Aerogel

Lan-Xing Li[1,#], Ying-Xian Li[1,#], Yu-Shuai Feng[1], Ya-Hao Du[1], Peng Ouyang [2], Qin Chen[2], Hui Yang[2], Huan Ye[1,*] and Fei-Fei Cao[1,*]

[1]College of Chemistry, Huazhong Agricultural University, Wuhan 430070 China

[2]State Key Laboratory of Material Processing and Die & Mould Technology, Department of Mechanics, School of Aerospace Engineering, Huazhong University of Science and Technology, Wuhan, Hubei 430074, China

E-mail: yehuan@mail.hzau.edu.cn, caofeifei@mail.hzau.edu.cn

[#]Equally contributed to this work.

# 1. Experimental

## 1.1 Fabrication of $Ti_3C_2T_x$ MXene

2 g of $Ti_3AlC_2$ powder was immersed in 40 ml of a 9 mol $L^{-1}$ HCl solution (Sinopharm Chemical Reagent Co., Ltd., Shanghai, China) containing 2 g of LiF (Sigma-Aldrich) at room temperature for 24 h. The obtained powder was washed several times with deionized water followed by ultrasonication for 1 hour. Then, the mixture was centrifuged at 3500 rpm for 60 min to remove larger particles. The supernatant yielded a black $Ti_3C_2T_x$ MXene colloid with a concentration of 4 mg $mL^{-1}$.

## 1.2 Fabrication of MXene membrane

1.5 mL of MXene suspension was added for vacuum filtration to attach MXene to the filter membrane. Following filtration, the membrane was washed several times with ethanol. The filter membrane containing the adhered MXene was removed and placed in a vacuum oven at 40°C to dry for 24 h. Then, the MXene membrane was carefully peeled away from the filter membrane.

## 1.3 Fabrication of carbon aerogel with oxygen-deficient $TiO_2$.

First, 500 mg of chitosan (CS) was dissolved in 75 mL of ultrapure water containing 50 mg of $CoCl_2 \cdot H_2O$ and 1000 μL of acetic acid. Then, 25 mL of a 4 mg $mL^{-1}$ MXene solution was added to the above mixture, followed by ultrasonic treatment and stirring for 60 min. Then, 10 mL of the resulting solution was transferred into a 55 mm×55 mm×45 mm plastic box, which was fixed on an open-lid iron box containing liquid nitrogen. The introduction of liquid nitrogen created a temperature gradient between the contact surface of the iron box and the surrounding air, promoting the growth of ice crystals that aligned horizontally and perpendicularly to the contact surface. The as-obtained sample was then freeze-dried to produce a chitosan-MXene aerogel (CM). Under an Ar atmosphere, the freeze-dried CM was subsequently annealed at 800°C for 2 h at a rate of 5°C $min^{-1}$, obtaining a carbonized, highly elastic aerogel with a chitosan/MXene weight ratio of 5:1.

## 1.4 Material characterization

X-ray diffraction (XRD) was performed using a Bruker D8 Advance instrument to

investigate the crystal structures of MXene and carbon aerogel. The scanning range was set from 5° to 80°, with a scanning rate of 2° $min^{-1}$, applying filtered Cu Kα radiation. Field emission scanning electron microscopy (SEM, SU-8000, acceleration voltage 10 kV), energy dispersive X-ray spectroscopy (EDS), and high-resolution transmission electron microscopy (HR-TEM) (JEOL-2100F) were used to characterize the surface morphologies, elemental distributions, and surface structures of MXene and carbon aerogel. X-ray photoelectron spectroscopy (XPS) measurements were conducted using an Escalab 250 XI (Thermo Scientific) equipped with 200 W monochromatic Al Kα radiation. Additionally, electron paramagnetic resonance (EPR) spectra were obtained using a Bruker EMXnano spectrometer. Time-of-flight secondary ion mass spectrometry (TOF-SIMS) measurements were conducted on an PHI nano TOF 3+ with a $Bi^{3+}$ ion source (30 kV, 10.5 nA), applying $Cs^+$ ion beam sputtering (2 keV) for depth profiling analysis over areas of 400 × 400 $\mu m^2$. X-ray microscopes (XRM) measurements were conducted using a Xradia 515 Versa (Zeiss) X-ray microscope. Mercury intrusion porosimetry (MIP) (MicroActive, AutoPore V 9600) tests were performed to characterize the porosity inside the carbon aerogel anode. $N_2$ adsorption–desorption isotherms was performed on a micromeritics (ASAP 2460, USA). The conductivity is measured using the RTS-9 type dual-electrode four-probe tester.

We further quantified the porosity of the carbon aerogel using mercury intrusion porosimetry (MIP). Our MIP instrument applies a maximum pressure of approximately 410 MPa, enabling us to measure pore diameters ranging from 0.003 μm to over 950 μm. The relationship between pore diameter and applied pressure is described by the Washburn equation: $Pr=-2\gamma\cos\theta$, where θ is the contact angle between mercury and the solid, and γ is mercury's surface tension. By maintaining constant θ and γ, we can gradually intrude mercury into smaller pores as pressure increases. Consequently, we record the intrusion volume (ΔV) per unit mass across different pore size intervals as pressure is incrementally increased. These measurements allow us to derive the overall porosity of 94.5% of the carbon aerogel.

**1.5 Electrochemical measurements**

CR2032-type coin cells were assembled in an argon-filled glove box ($O_2$ < 0.1 ppm, $H_2O$ < 0.1 ppm). The separator used was a polypropylene film (Celgard, 2400). The electrolyte consisted of a 1:1 volumetric mixture of 1,3-dioxolane (DOL) and dimethoxyethane (DME), containing 1 M lithium bis(trifluoromethanesulfonyl)imide (LiTFSI) with 1 wt.% $LiNO_3$. For testing Coulombic efficiency, carbon aerogel was used as the working electrode and lithium foil as the counter electrode. For symmetric cell testing, each cell was first cycled three times between 0.01 and 1 V (versus $Li^+/Li$) to stabilize the solid electrolyte interphase (SEI) layer. Then, they were pre-plated with 6 mA h $cm^{-2}$ of Li, disassembled and reassembled into symmetric cells. For full cell testing, LFP (Taiwan ALeees) cathodes were fabricated using LFP active material mixed with Super P and polyvinylidene fluoride (PVDF, Alfa Aesar) in a weight ratio of 8:1:1, with N-methyl-2-pyrrolidone (NMP, Sigma-Aldrich) used as the solvent. The LFP loading was about 15 mg $cm^{-2}$, while the pouch cell had an LFP loading of about 8 mg $cm^{-2}$. The galvanostatic charge/discharge performance of the full cells was tested on a NEWARE testing system with a voltage range of 2.0-4.0 V at 0.5 and 1 C. Electrochemical impedance spectra (EIS) were performed using a CHI-760E electrochemical workstation (CH Instruments, Inc., Shanghai) across a frequency range of 0.1 Hz to 100 kHz. CV curves of the half cells were recorded in the voltage range of −0.2 to 1.0 V at a scan rate of 2 mV $s^{-1}$. The lithium-ion diffusion coefficient is fitted using variable sweep CV. The galvanostatic intermittent titration technique (GITT) was conducted on Li|Li symmetric cells at a current density of 0.5 mA $cm^{-2}$ with a Li deposition of 0.5 mA h $cm^{-2}$. Tafel plots were obtained from Li symmetric cells by scanning the voltage from −0.2 to 0.2 V at a scan rate of 1 mV $s^{-1}$ and a current density of 1 mA $cm^{-2}$ with a deposition of 1 mA h $cm^{-2}$ after 25 cycles.

To reduce initial irreversible lithium loss and enhance the electrochemical performance of the full cell, the carbon aerogel anode was pre-lithiated using an electrochemical method for three cycles. This involved assembling a half-cell with the carbon aerogel as the working electrode and lithium metal foil serving as both the counter and reference electrodes. The voltage range was set at 0.01–1 V, and the half-cell was galvanostatically discharged at a constant current density of 0.5 mA $cm^{-2}$ until

a total lithium capacity of 6 mAh cm$^{-2}$ was deposited onto the carbon aerogel electrode. For the low N/P full cell test, The LFP loading was about 23 mg cm$^{-2}$ (3.5 mA h cm$^{-2}$). After completely extracting of Li from the $LiFePO_4$, the delithiated lithium iron phosphate, that is, iron phosphate ($FePO_4$) electrode was disassembled in the glove box served as the cathode of the full cell. The carbon aerogel anode deposited 3.85 mA h cm$^{-2}$ of Li is regarded as the anode of the full cell, corresponds to a Li utilization rate of 90%. Then we coupled the carbon aerogel/Li anode with the delithiated lithium iron phosphate cathode to assemble a Li-metal full cell, the N/P ratio is about (3.85-3.50)/3.50=0.1. For the condition of lean electrolyte full cell test, the LFP loading was about 15-16 mg cm$^{-2}$ (based on a 10 mm diameter electrode), we significantly reduced the electrolyte amount from 45 μL to 25 μL per cell, corresponding to a decreased E/C ratio from 3.8 to 2.0 μL mg$^{-1}$.

**1.6 Atomistic-scale calculation details**

All the spin-polarized first-principles calculations were performed via the Vienna ab initio simulation package (VASP)[1]. The ion-electron interactions were described by the projector augmented wave (PAW) method, and the generalized gradient approximation (GGA) with the Perdew-Burke-Ernzerhof (PBE) was used to treat the interaction of electronic exchange-correlation energy[2–5]. The Monkhorst-Pack k-point mesh of 6x6x1 was set in this work. The cutoff energy for plane-wave is 520 eV. The convergence threshold is $10^{-6}$ eV and $1\times10^{-3}$ eV/Å for energy and force, respectively. The van der Waals interaction was treated by Grimme scheme (DFT-D3)[5].

The structures of $TiO_2$ (101) and $V_o$-$TiO_2$ (101) surface were built, where the vacuum space along the z direction is set to be 18 Å, which is enough to avoid interaction between the two neighboring images. The selection of lithium adsorption sites was determined by the coordination environment of surface oxygen atoms. For the pristine $TiO_2$ surface, surface oxygen atoms with fourfold coordination are identified as potential adsorption sites. The underlying reason is that a higher coordination number can stabilize the adsorbed lithium atom through multiple lithium-oxygen interactions, resulting in more favorable adsorption energies. In the case of the $TiO_2$ surface with oxygen vacancies, we considered threefold coordinated oxygen atoms neighboring

these vacancy sites as potential adsorption sites for lithium.

The all atoms were relaxed adequately to remove the internal stress of systems. Then, one Li atom was adsorbed on surface (see atomic structures).

Adsorption energy $\Delta E_{abs}$ of Li atom on the surface of substrates was defined as[6]:

$$\Delta E_{ads} = E_{*Li} - (E_{*} + \mu_{Li}) \quad (1)$$

where *Li and * denote the adsorption of Li atom on substrates and the bare substrates, $\mu_{Li}$ denotes the energy of per Li atom in its bulk.

The embedded energy $\Delta E_{emb}$ of Li atom in the substrates was defined as:

$$\Delta E_{emb} = E_{*Li\text{-}int} - (E_{*} + \mu_{Li}) \quad (2)$$

where, $E_{*Li\text{-}int}$ denoted the total energy of Li in substrates.

For the calculation of Li migration energy barrier on the surface of arch-structured carbon aerogels under tensile stress/strain, LAMMPS based molecular dynamics simulations were conducted with ReaxFF potential. In the simulation, a single layer graphene sheet was adopted to mimic the surface layer of the carbon aerogels. By applying different levels of tensile strain in the armchair direction of the graphene sheet, a Li atom was first put on the top of a hollow site that far away from the edge of the graphene sheet, and then moved to the other nearby hollow sites gradually, with the energy landscapes along the moving paths recorded[7,8]. According to the energy landscape along the minimum energy path, the corresponding energy barriers were then calculated and the energy barrier versus strain curve was plotted.

**1.7 Finite element calculation details**

To simulate deformation and stress of an arch-structured carbon aerogel under compressive loading, a 3D Sine wave-shaped carbon aerogel structure was constructed in COMSOL Multiphysics. The structure was placed on a rigid substrate and the top of it was compressed via a rigid plate. Periodical boundary conditions were applied on the other four lateral surface. By assigning the structure with elastic properties, the stress distributions in structure were simulated with the Solid Mechanics module. For comparison, a planar structure subjected to compression was also simulated.

## 2. Supplementary Figures

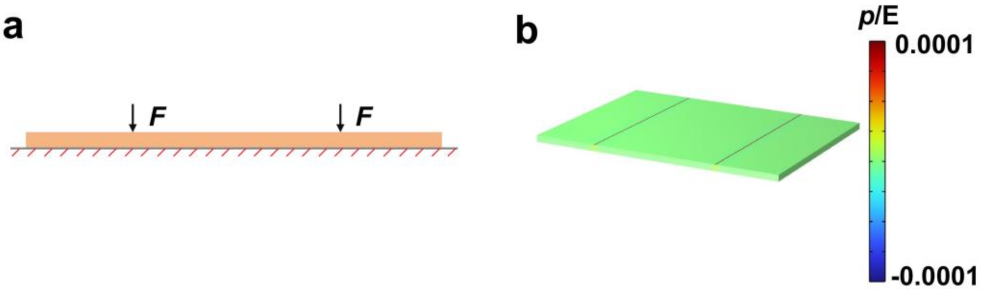


**Figure S1.** a) Schematic diagram of stress analysis of a planar structure under compressive loading. b) Finite element simulation for pressure distribution of the planar structure subjected to compressive loading, showing no large-scale stress gradient generated.

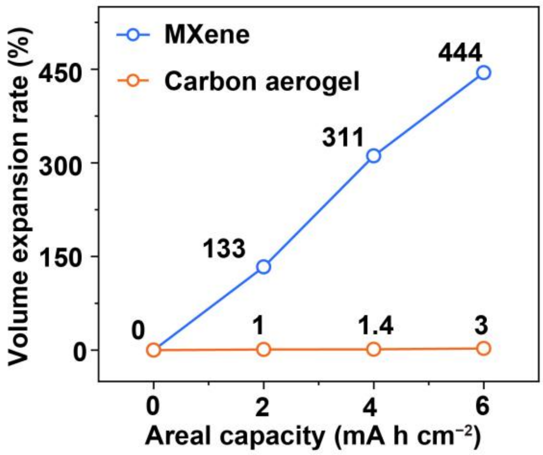


**Figure S2.** Electrode volume expansion rate of Li-carbon aerogel anode after plating various areal capacities of Li.

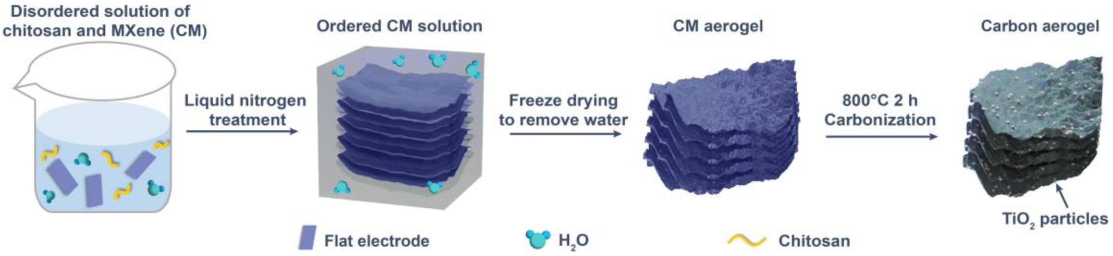


**Figure S3.** Schematic diagram of the synthesis process of carbon aerogel.

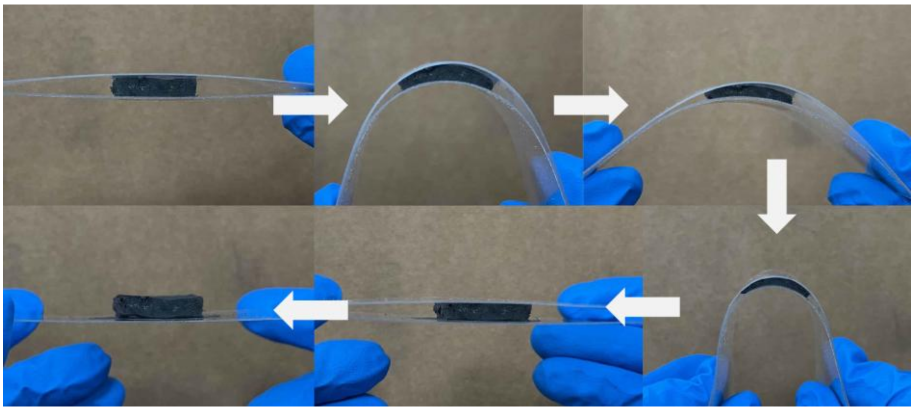

**Figure S4.** Optical images of carbon aerogel bending test. Carbon aerogel retains its structural integrity after being bent to smaller angles.

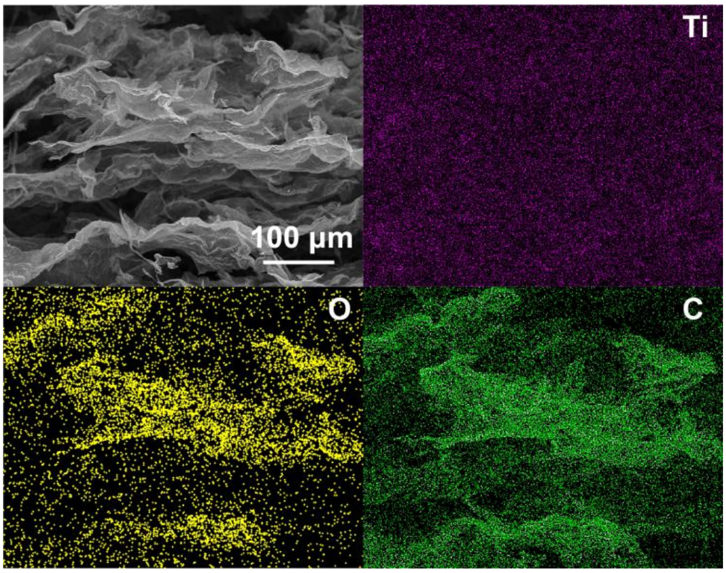


**Figure S5.** EDS elemental mapping images of the carbon aerogel.

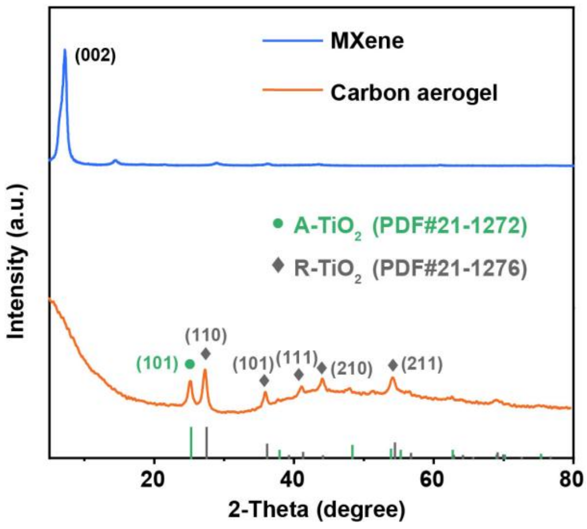


**Figure S6.** XRD patterns of MXene and carbon aerogel.

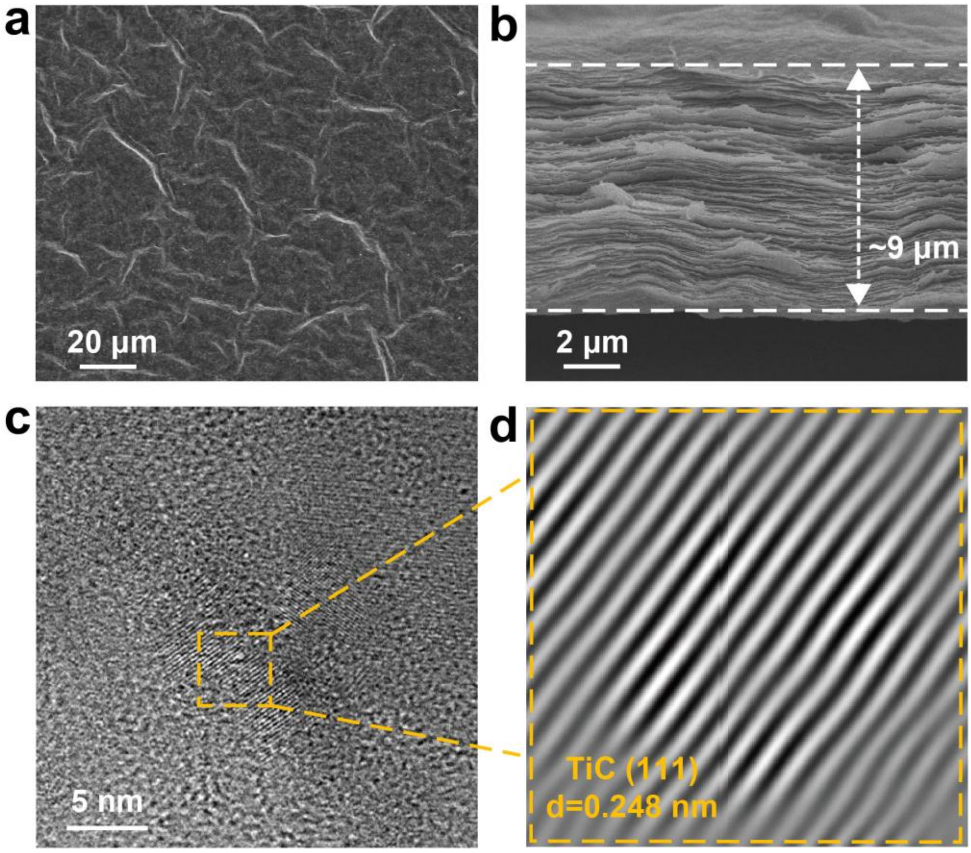


**Figure S7.** (a) Top-view and (b) cross-sectional SEM images of MXene without Li metal plating. HRTEM image of MXene (c) and corresponding local magnification (d).

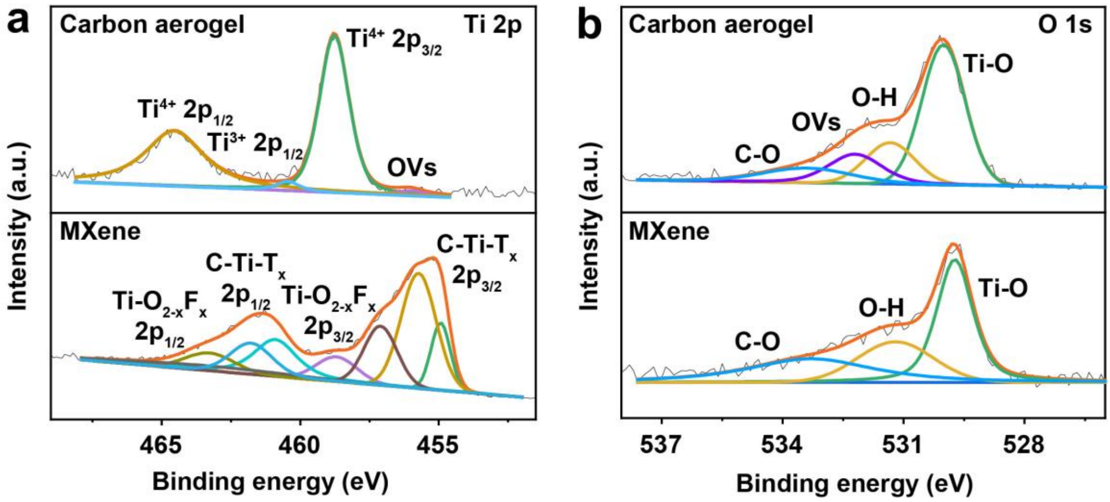


**Figure S8.** (a) Ti 2p and (b) O 1s XPS profiles of carbon aerogel and MXene before cycling.

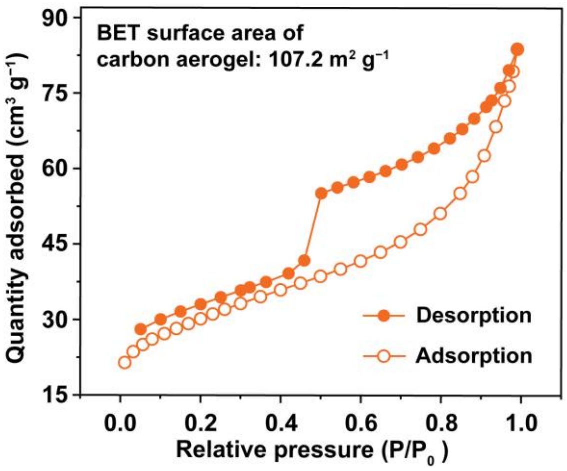


**Figure S9.** $N_2$ adsorption/desorption isotherm of carbon aerogel.

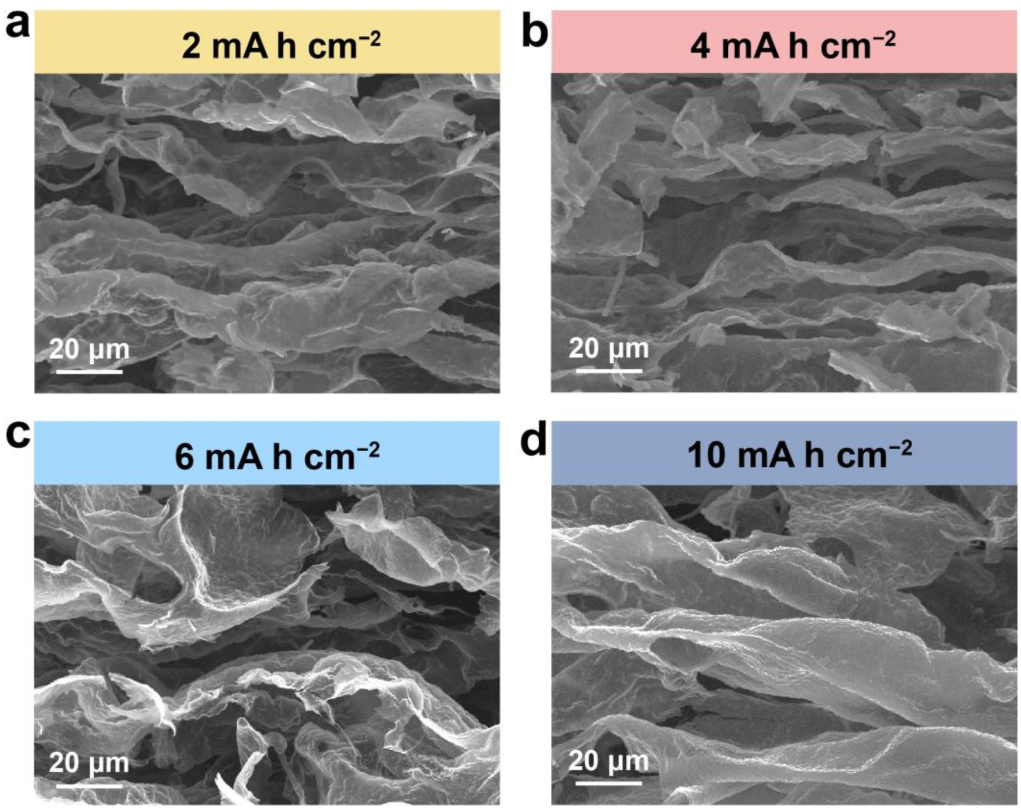


**Figure S10.** SEM images of carbon aerogel after being plated with (a) 2 mA h $cm^{-2}$, (b) 4 mA h $cm^{-2}$, (c) 6 mA h $cm^{-2}$, and (d) 10 mA h $cm^{-2}$ of Li metal at 0.5 mA $cm^{-2}$.

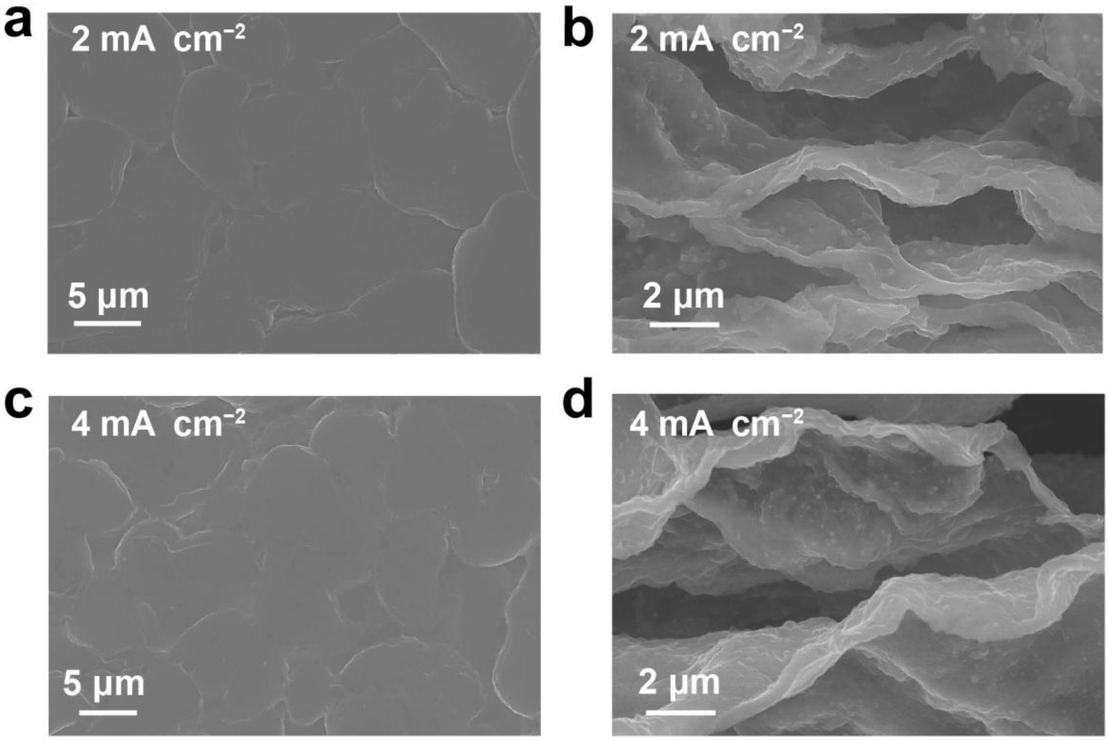


**Figure S11.** (a, c) Top-view SEM images of carbon aerogel after being plated with 6 mA h cm$^{-2}$ of Li metal at a high current density of 2 and 4 mA cm$^{-2}$, respectively. (b, d) Cross-section SEM images of carbon aerogel after being plated with 6 mA h cm$^{-2}$ of Li metal at a high current density of 2 and 4 mA cm$^{-2}$, respectively.

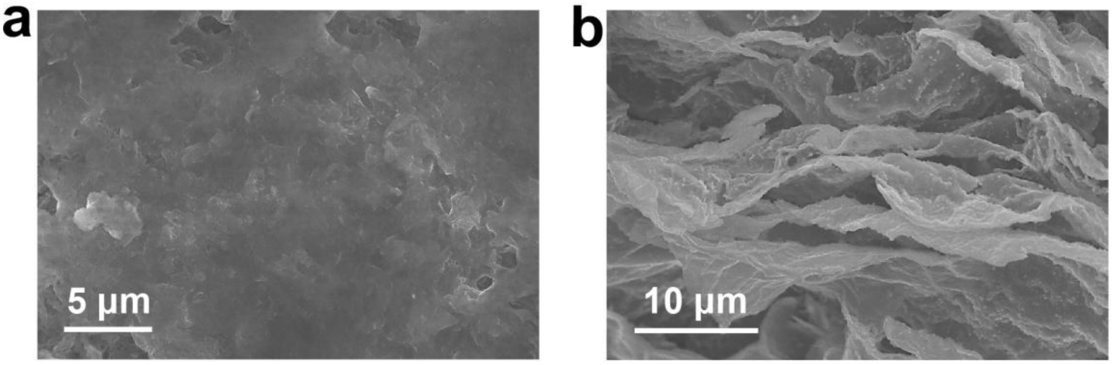


**Figure S12.** (a) Top-view and (b) Cross-section SEM images of carbon aerogel after being plated with 6 mA h $cm^{-2}$ of Li metal at 1 mA $cm^{-2}$ in ester carbonate electrolyte.

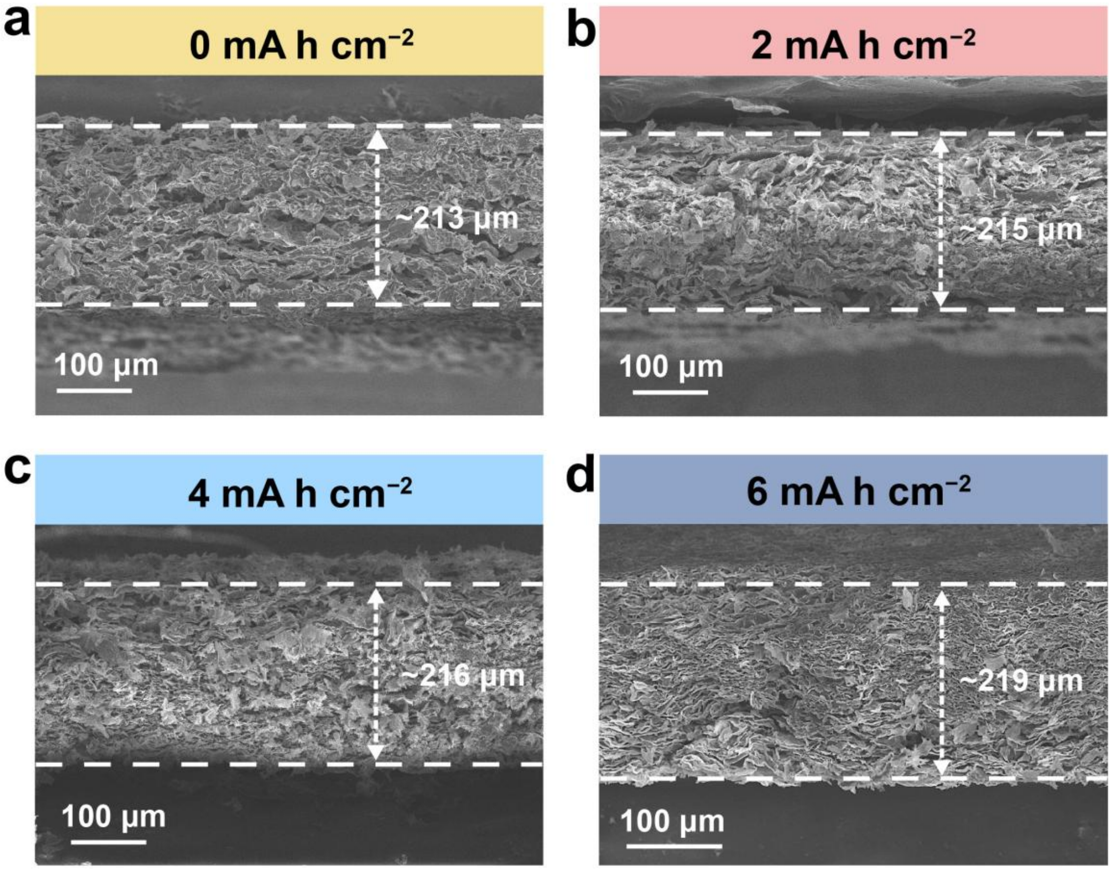


**Figure S13.** Side-view SEM images of carbon aerogel after being plated with (a) 0 mA h cm$^{-2}$, (b) 2 mA h cm$^{-2}$, (c) 4 mA h cm$^{-2}$, and (d) 6 mA h cm$^{-2}$ of Li metal at 0.5 mA cm$^{-2}$.

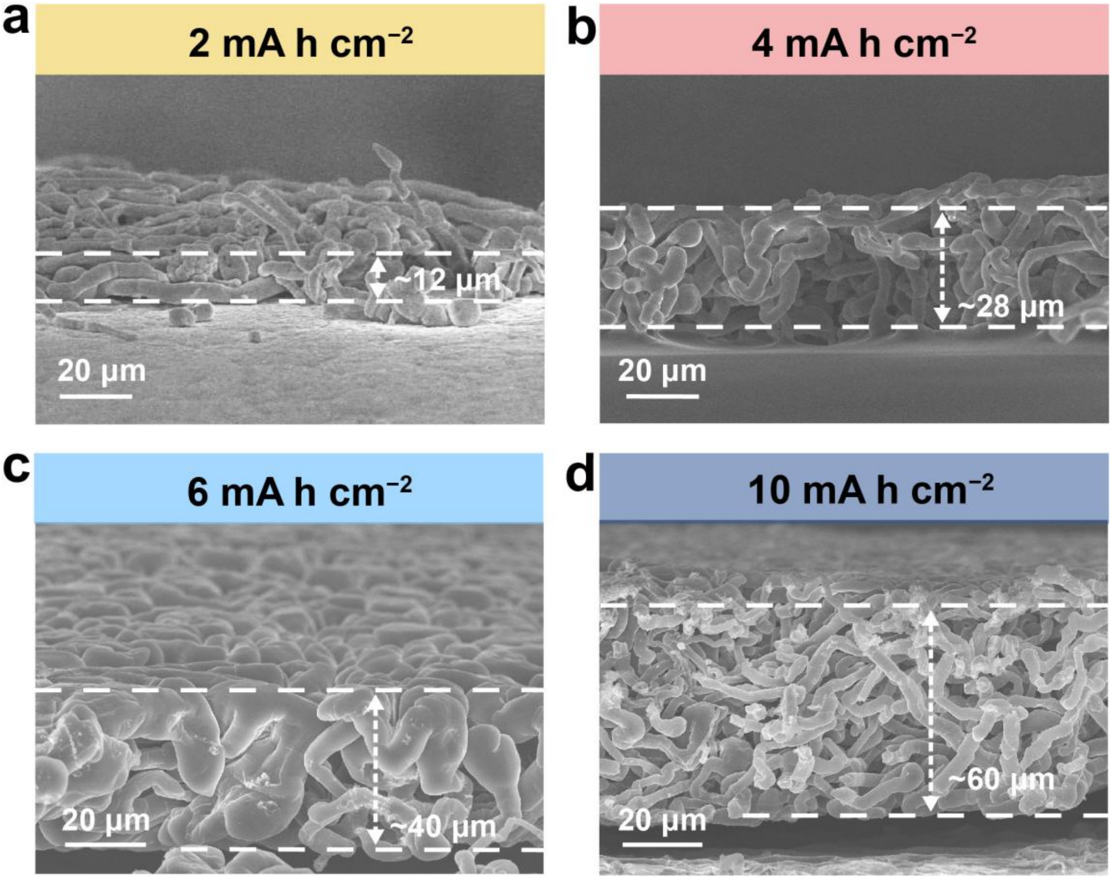


**Figure S14.** Side-view SEM images of MXene after being plated with (a) 2 mA h cm$^{-2}$, (b) 4 mA h cm$^{-2}$, (c) 6 mA h cm$^{-2}$, and (d) 10 mA h cm$^{-2}$ of Li metal at 0.5 mA cm$^{-2}$.

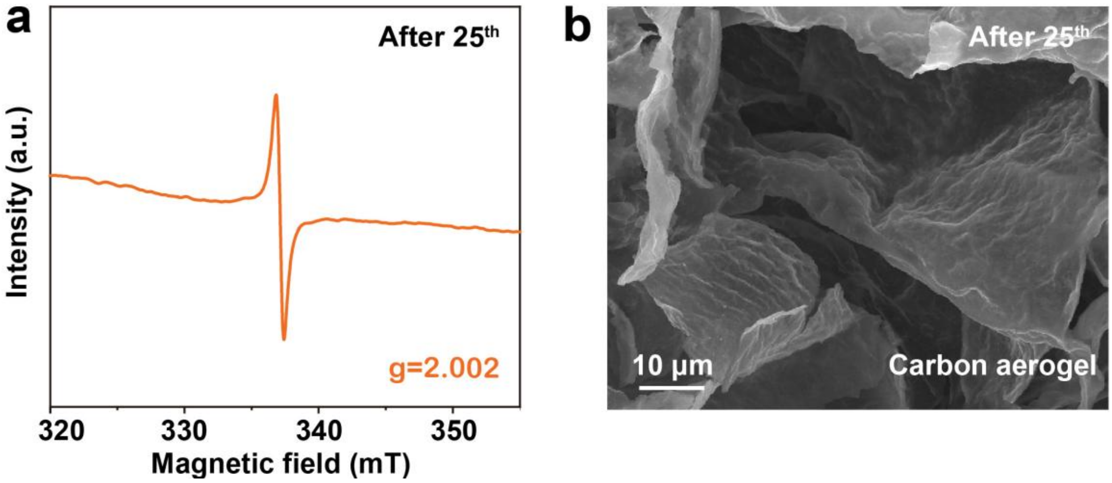


**Figure S15.** (a) EPR spectra of Li|carbon aerogel half cells after 25 cycles. The current density is 1 mA cm$^{-2}$ and the areal capacity is 6 mA h cm$^{-2}$. (b) SEM image of the carbon aerogel and MXene-based Li|Li symmetric cells after 25 cycles. The current density is 1 mA cm$^{-2}$ and the areal capacity is 1 mA h cm$^{-2}$.

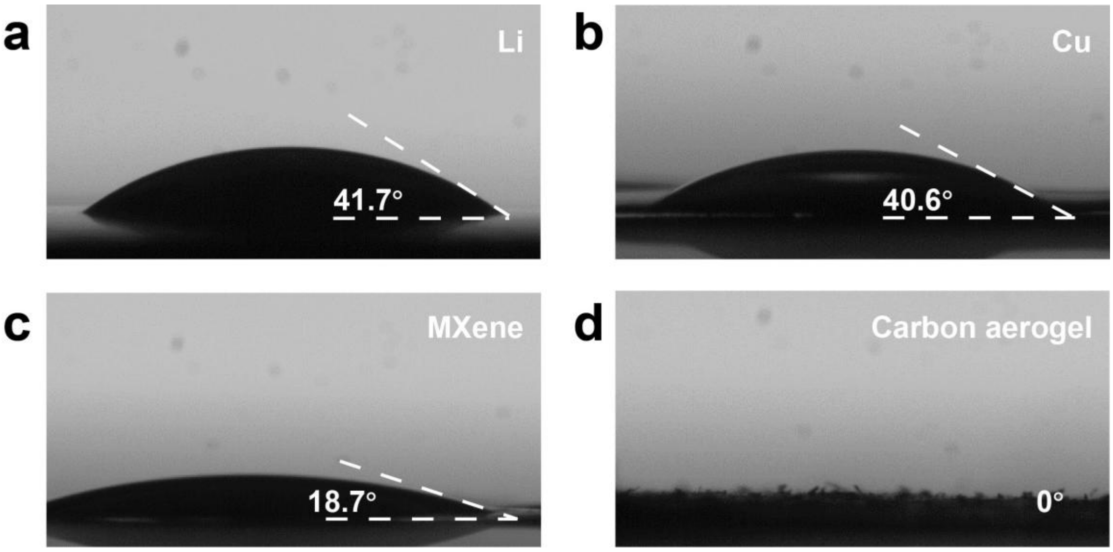


**Figure S16.** Contact angle measurements on different current collector surfaces with 1 M LiTFSI+DOL/DME (v/v=1:1) +1% $LiNO_3$ electrolyte.

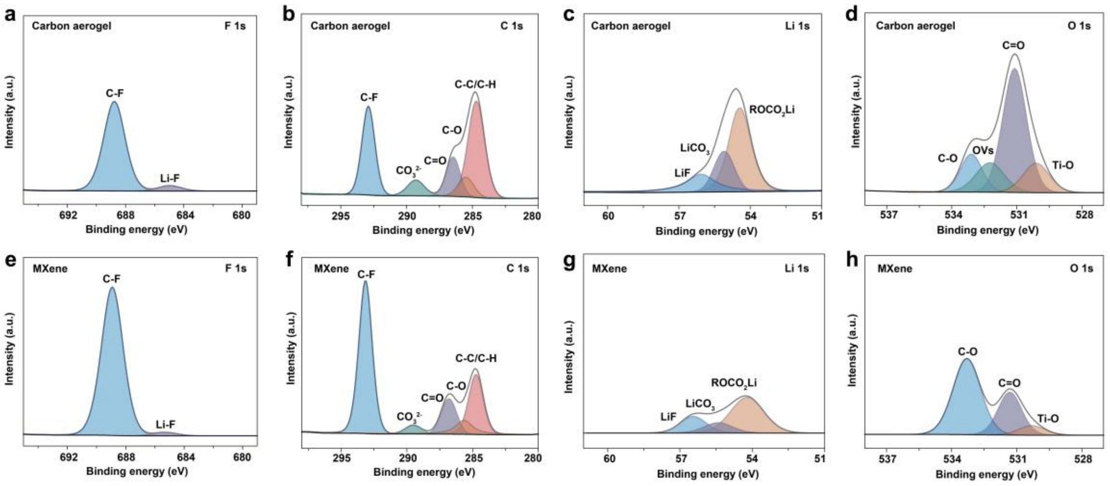


**Figure S17.** XPS spectra of the carbon aerogel and MXene after 10 plating/stripping cycles at 2 mA cm$^{-2}$ and 2 mA h cm$^{-2}$. (a) F 1s, (b) C 1s, (c) Li 1s and (d) O 1s spectrum profiles for the cycled carbon aerogel. (e) F 1s, (f) C 1s, (g) Li 1s and (h) O 1s spectrum profiles for the cycled MXene.

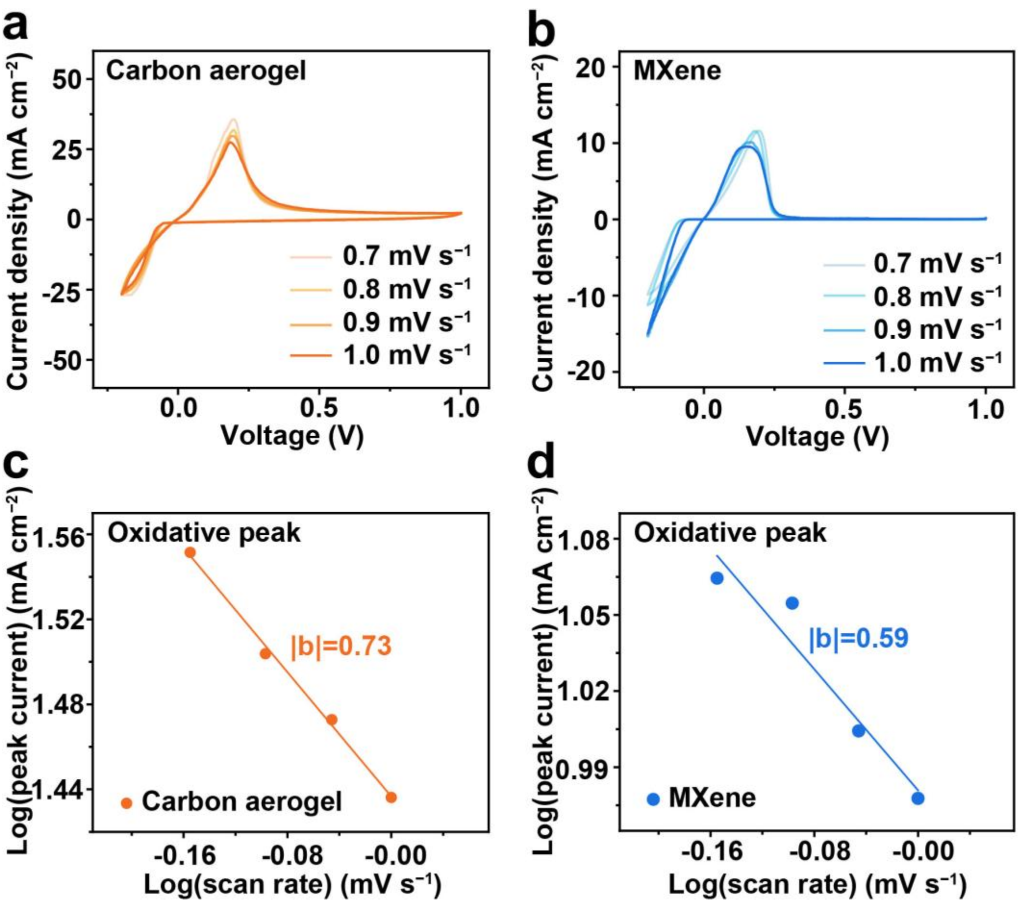


**Figure S18.** Cycle voltammetry of (a) carbon aerogel|Li and (b) MXene|Li half-cells at scan rates of 0.7~1 mV $s^{-1}$. Linear fitting of log (oxidative peak current) to log (scan rate) from the CV curves at different scan rates of cells with (c) carbon aerogel and (d) MXene.

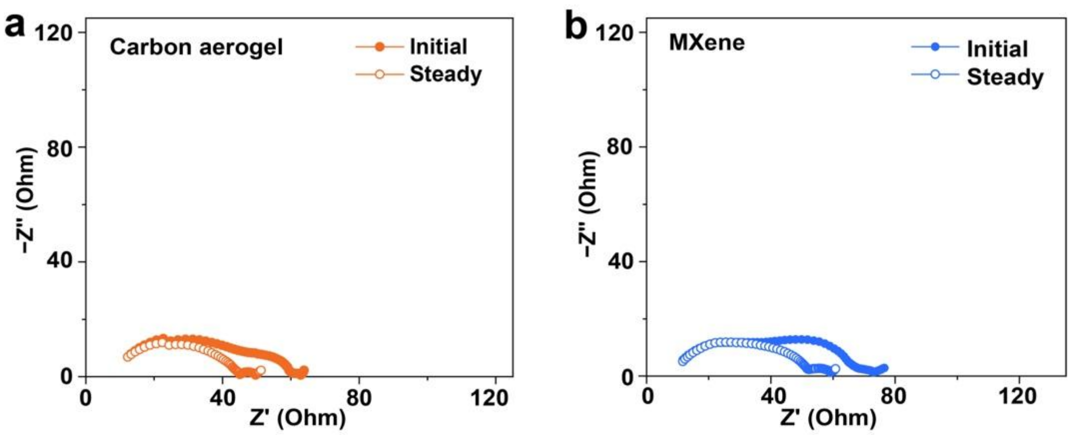


**Figure S19.** The corresponding electrochemical impedance spectra (EIS) of (a) carbon aerogel and (b) MXene-based Li|Li symmetric cells before and after polarization.

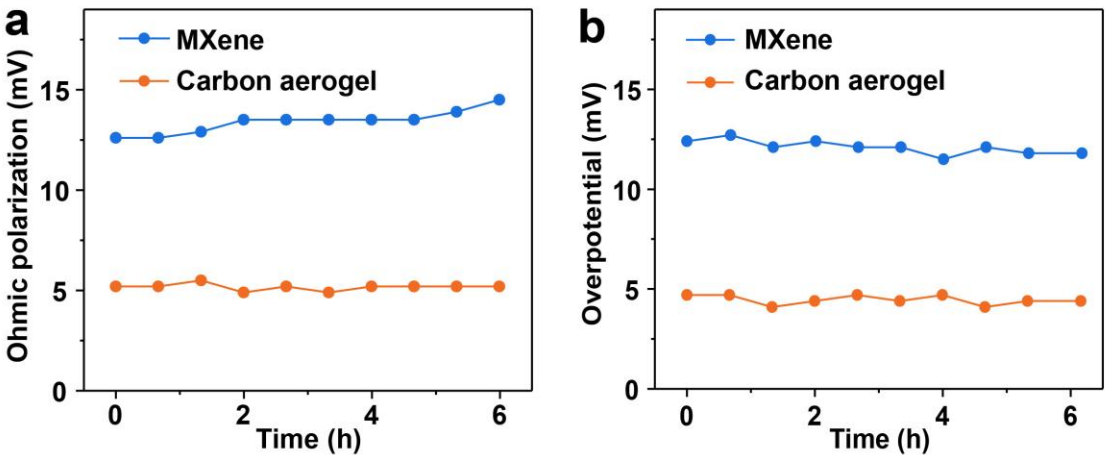


**Figure S20.** (a) Ohmic polarization and (b) overpotential evolution fitted from GITT plots in Figure 4i for carbon aerogel and MXene-based Li|Li symmetric cells.

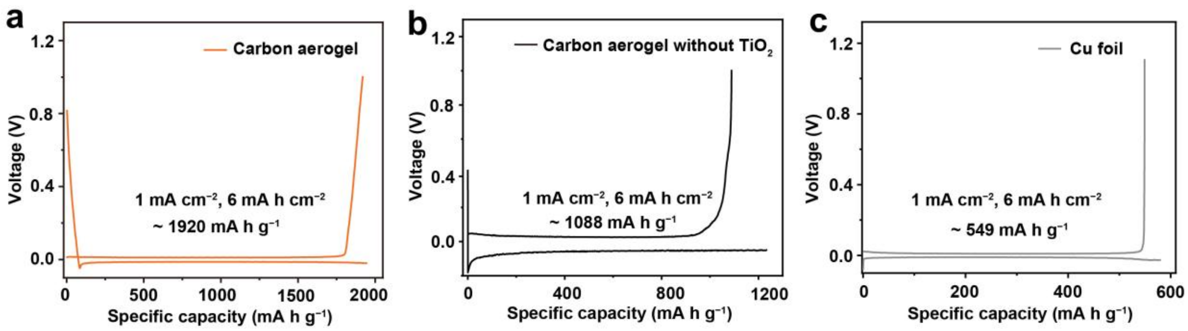


**Figure S21.** Charge-discharge curves of (a) carbon aerogel|Li, (b) Carbon aerogel without $TiO_2$|Li half-cells and (c) Li|Cu half-cells.

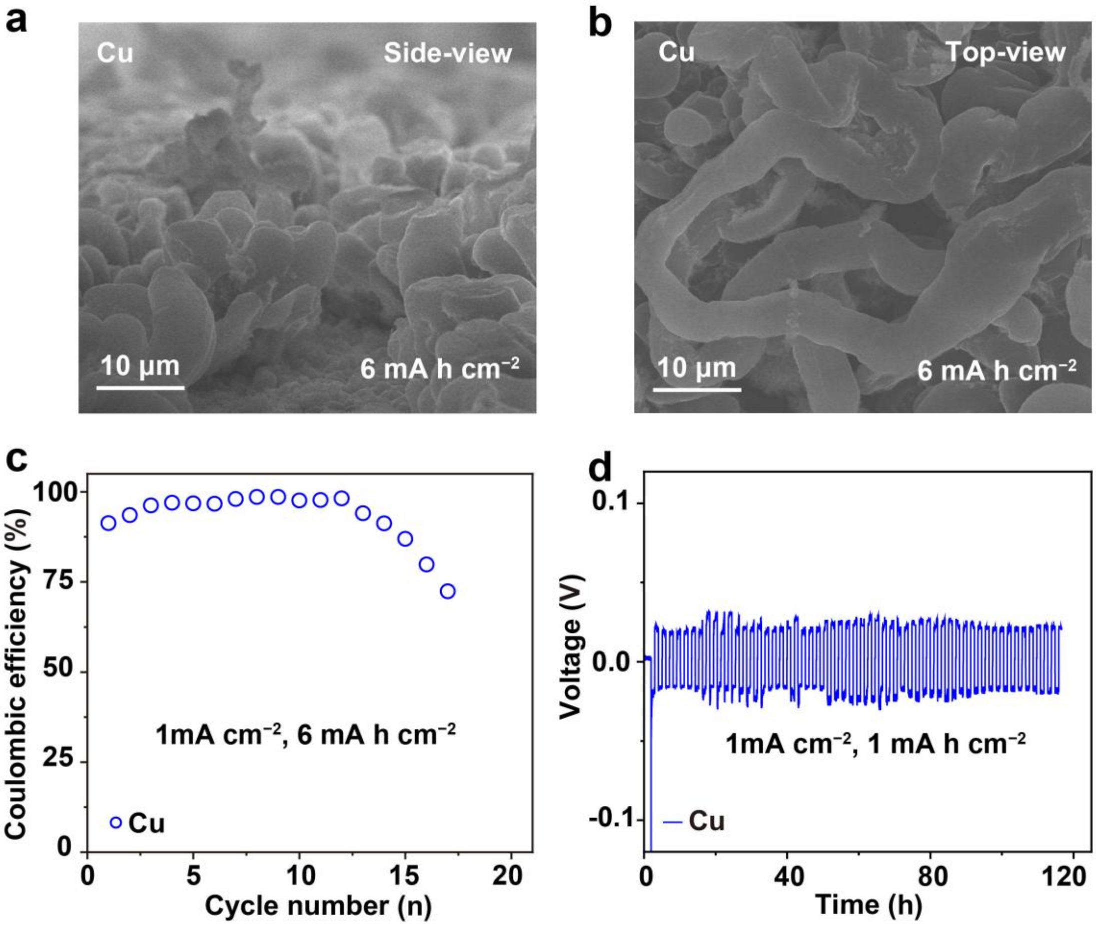


**Figure S22.** (a) Top-view SEM image of Cu foil after being plated with 6 mA h cm$^{-2}$ of Li metal at 1 mA cm$^{-2}$. (b) Side-view SEM image of Cu foil after being plated with 6 mA h cm$^{-2}$ of Li metal at 1 mA cm$^{-2}$. (c) Coulombic efficiency of Cu foil at 1 mA cm$^{-2}$ with a Li plating capacity of 6 mA h cm$^{-2}$. (d) Voltage profile of Cu foil-based Li|Li symmetric cell with a Li plating capacity of 1 mA h cm$^{-2}$ at 1 mA cm$^{-2}$.

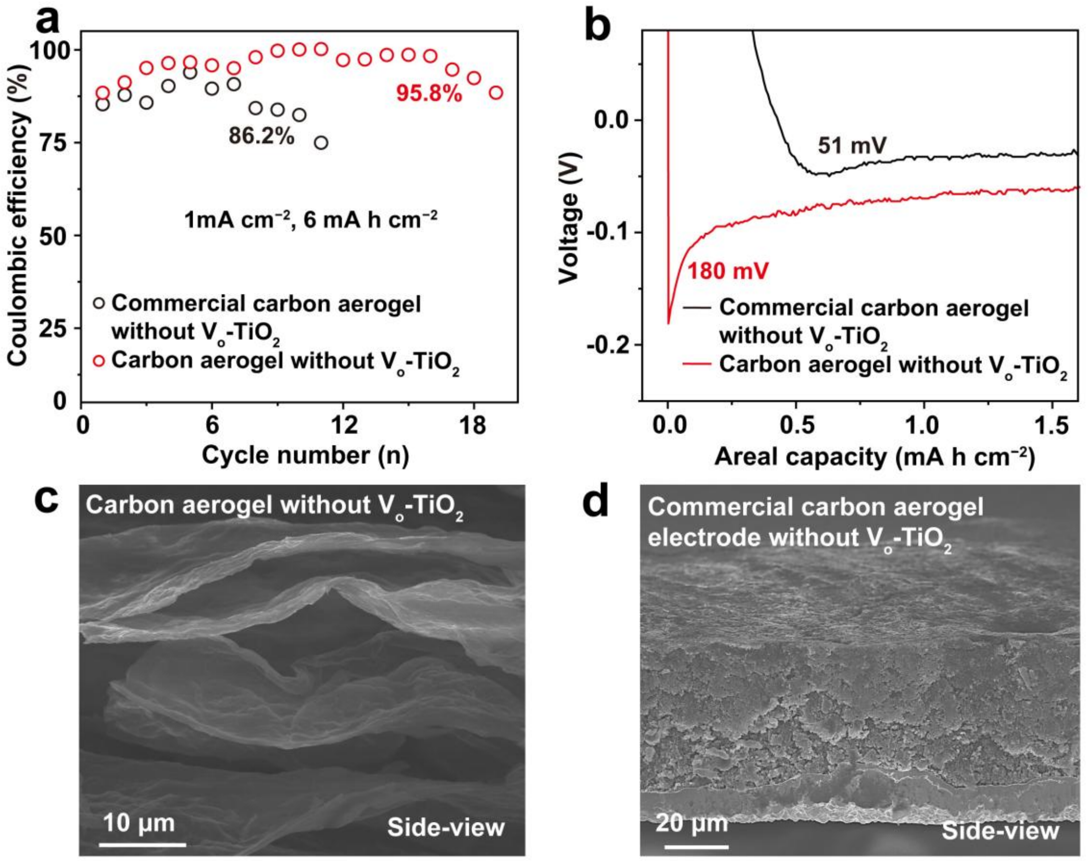


**Figure S23.** (a) Coulombic efficiencies of commercial carbon aerogel electrode without $V_o$-$TiO_2$ and carbon aerogel without $V_o$-$TiO_2$ at 1 mA cm$^{-2}$ with a Li plating capacity of 6 mA h cm$^{-2}$. (b) Corresponding voltage profiles of commercial carbon aerogel electrode without $V_o$-$TiO_2$ and carbon aerogel without $V_o$-$TiO_2$. Side-view SEM images of the (c) carbon aerogel without $V_o$-$TiO_2$ and (d) commercial carbon aerogel electrode without $V_o$-$TiO_2$.

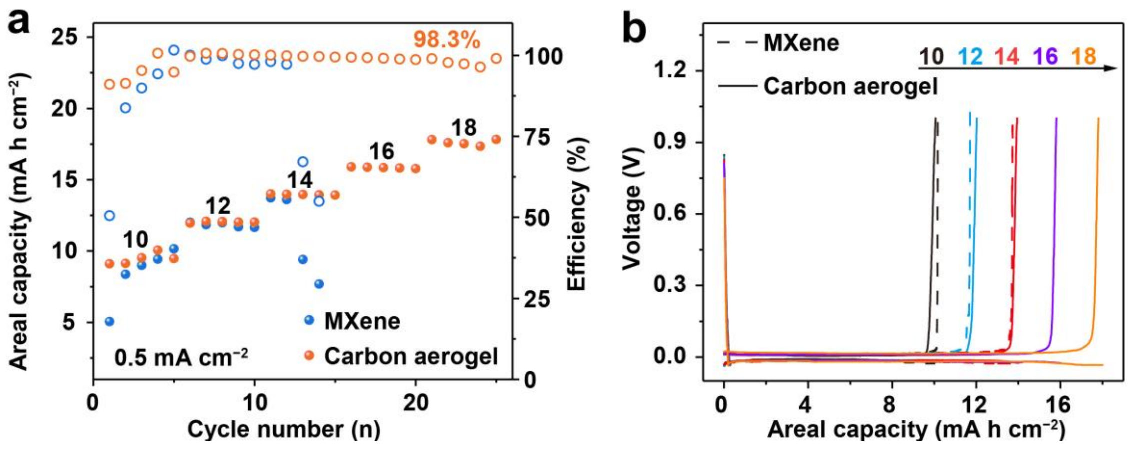


**Figure S24.** (a) Coulombic efficiency and (b) plating/stripping voltage profiles of carbon aerogel and MXene with a different Li plating capacity at 0.5 mA $cm^{-2}$.

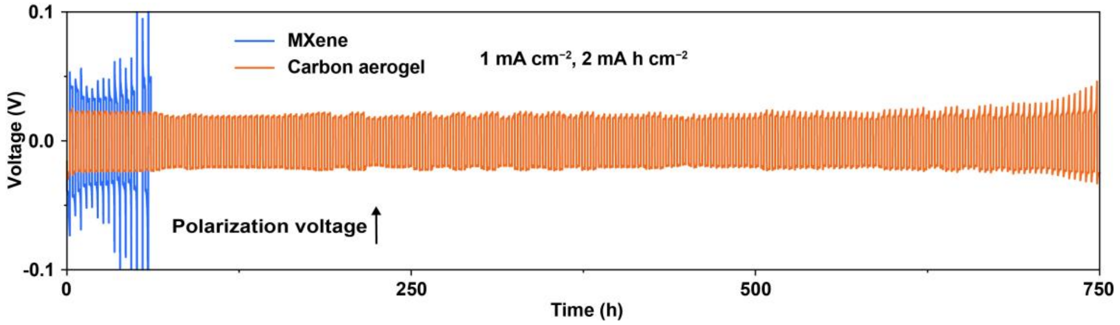


**Figure S25.** Voltage profiles of carbon aerogel and MXene-based Li|Li symmetric cells with a Li plating capacity of 2 mA h cm$^{-2}$ at 1 mA cm$^{-2}$.

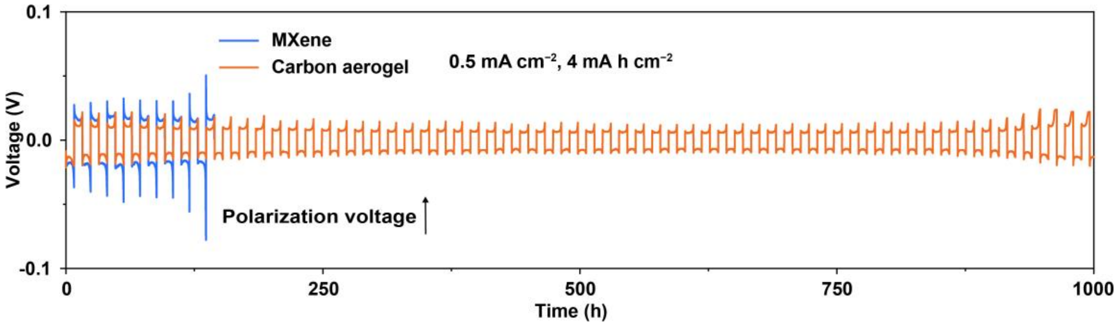


**Figure S26.** Voltage profiles of carbon aerogel and MXene-based Li|Li symmetric cells with a Li plating capacity of 4 mA h cm$^{-2}$ at 0.5 mA cm$^{-2}$.

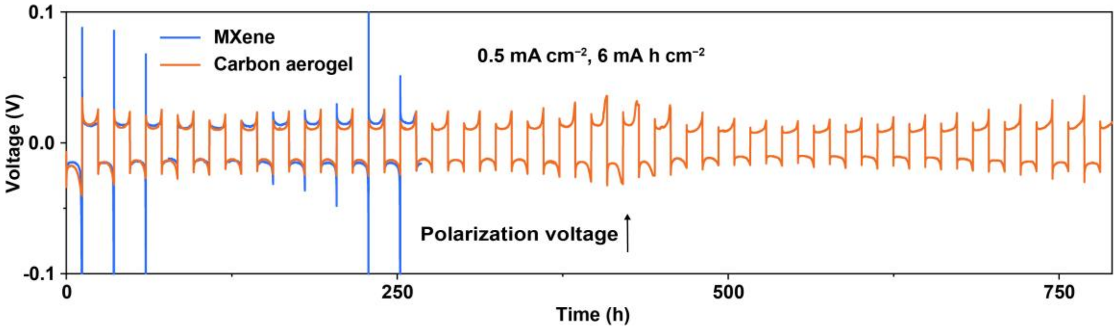


**Figure S27.** Voltage profiles of carbon aerogel and MXene-based Li|Li symmetric cells with a Li plating capacity of 6 mA h cm$^{-2}$ at 0.5 mA cm$^{-2}$.

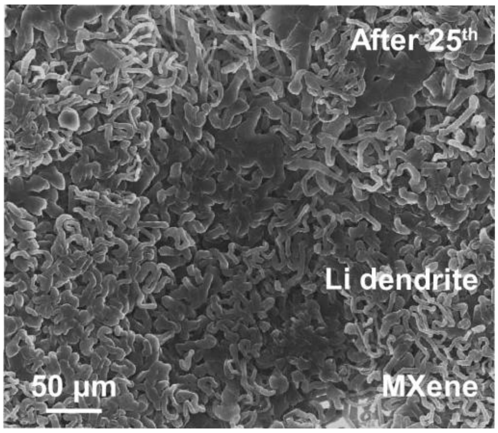


**Figure S28.** Top-view SEM image of the carbon aerogel and MXene-based Li|Li symmetric cells after 25 cycles. The current density is 1 mA cm$^{-2}$ and the areal capacity of Li metal is 1 mA h cm$^{-2}$.

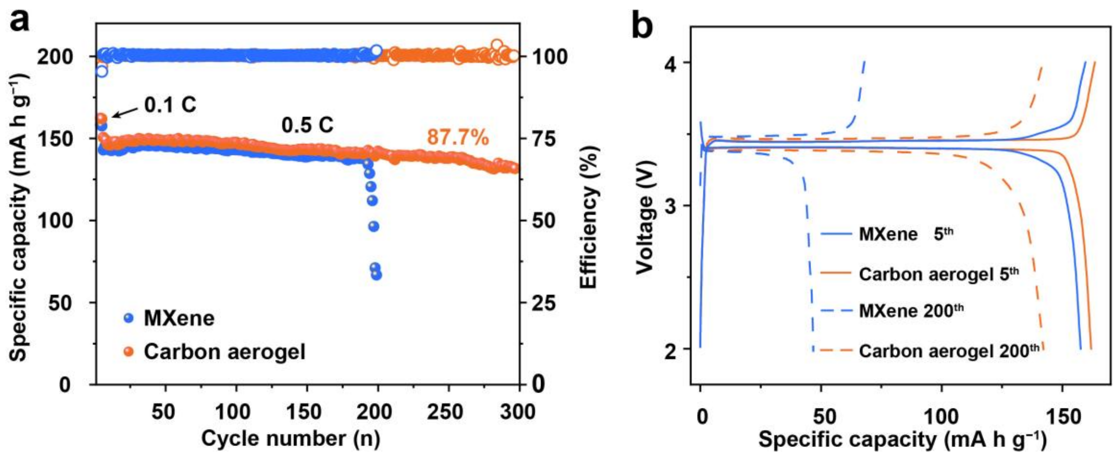


**Figure S29.** (a) The cycling performance at 0.5 C in the voltage range of 2.0-4.0 V and (b) Charge-discharge curves of carbon aerogel/Li|LFP and MXene/Li|LFP full cells.

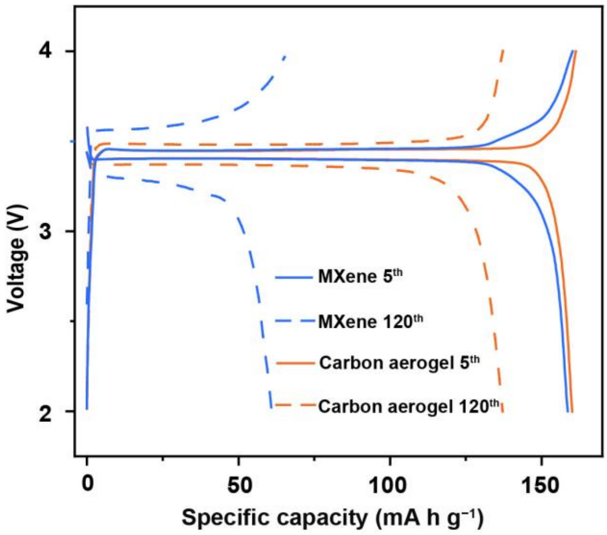


**Figure S30.** Charge-discharge curves of carbon aerogel/Li|LFP and MXene /Li|LFP full cells at 1 C in the voltage range of 2.0-4.0 V.

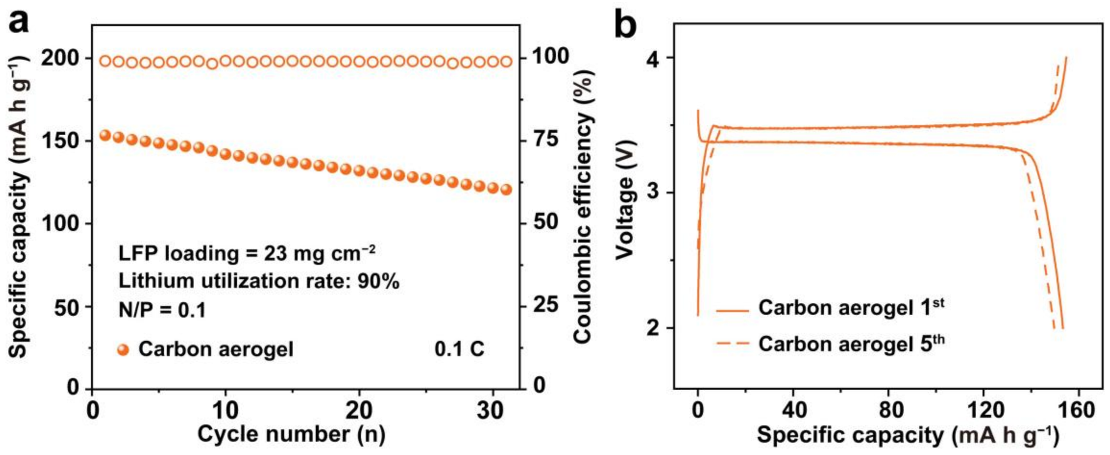


**Figure S31.** (a) The cycling performance of carbon aerogel/Li|LFP with an N/P ratio of approximately 0.1 at 0.1 C in the voltage range of 2.0-4.0 V and (b) corresponding Charge-discharge curves for $1^{st}$ and $5^{th}$ cycle.

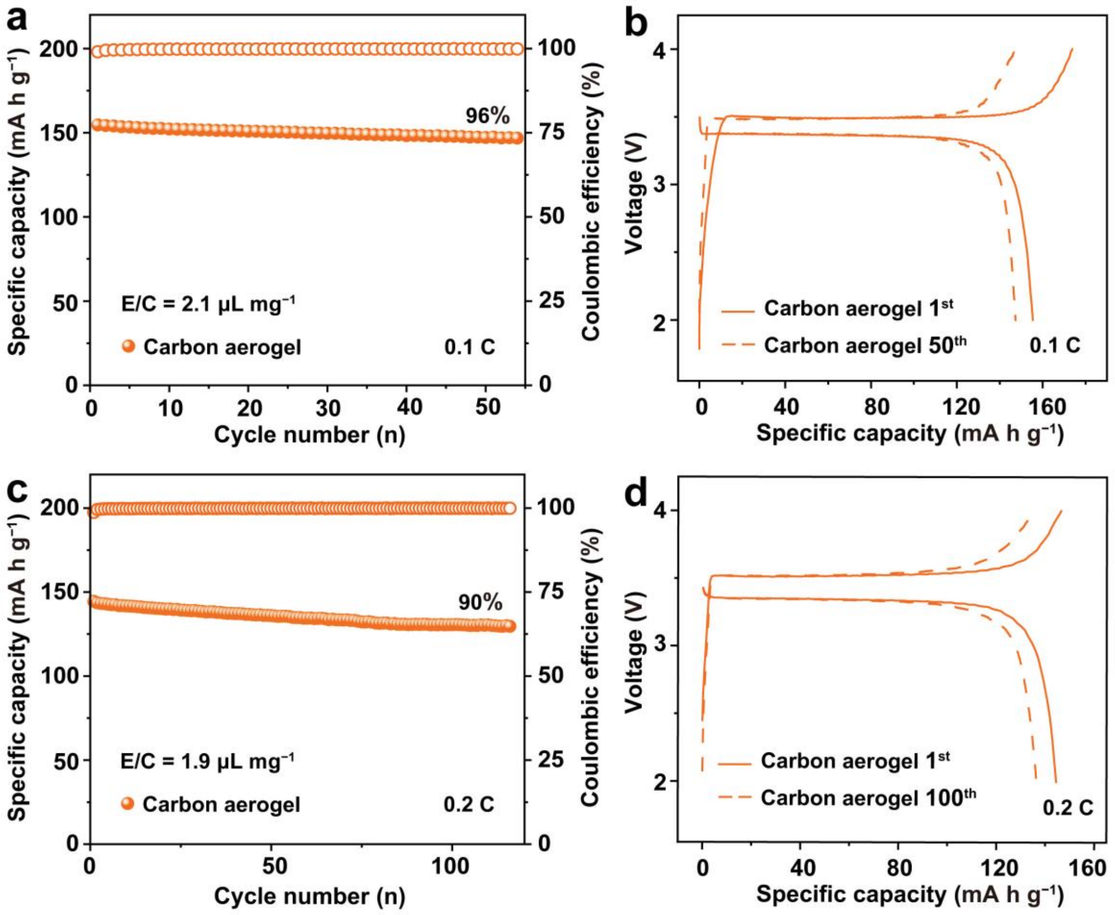


**Figure S32.** (a) The cycling performance of carbon aerogel/Li|LFP with a E/C ratio of 2.1 at 0.1 C in the voltage range of 2.0-4.0 V and (b) corresponding Charge-discharge curves. (c) The cycling performance of carbon aerogel/Li|LFP with a E/C ratio of 1.9 at 0.2 C in the voltage range of 2.0-4.0 V and (d) corresponding Charge-discharge curves.

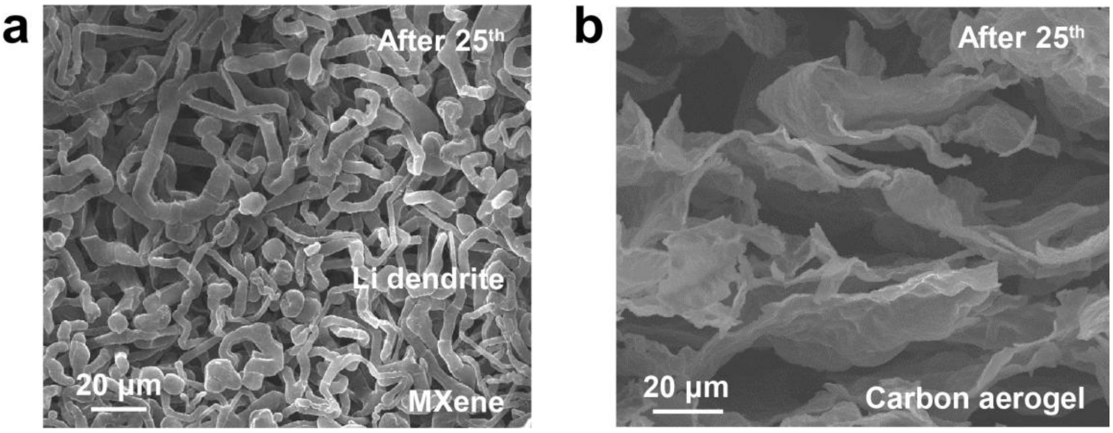


**Figure S33.** (a) Top-view SEM images of MXene/Li electrode of MXene/Li|LFP full cell in discharged state after 25 cycles at 1 C. (b) cross-sectional image of the carbon aerogel/Li electrode of carbon aerogel/Li|LFP full cell in discharged state after 25 cycles at 1 C.

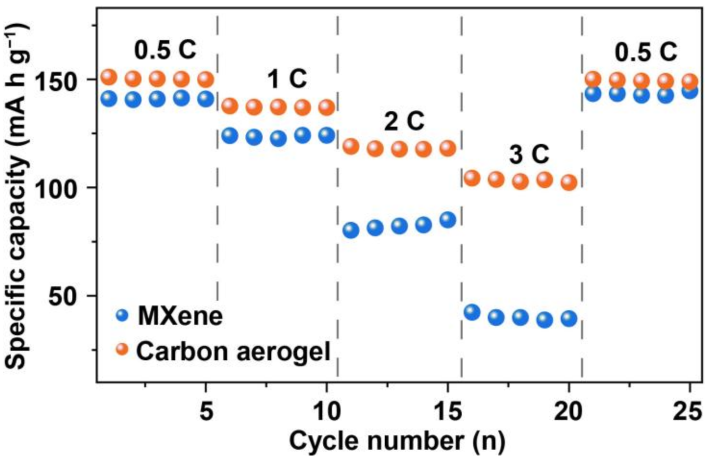


**Figure S34.** Rate capability of carbon aerogel/Li|LFP and MXene/Li|LFP at different current densities.

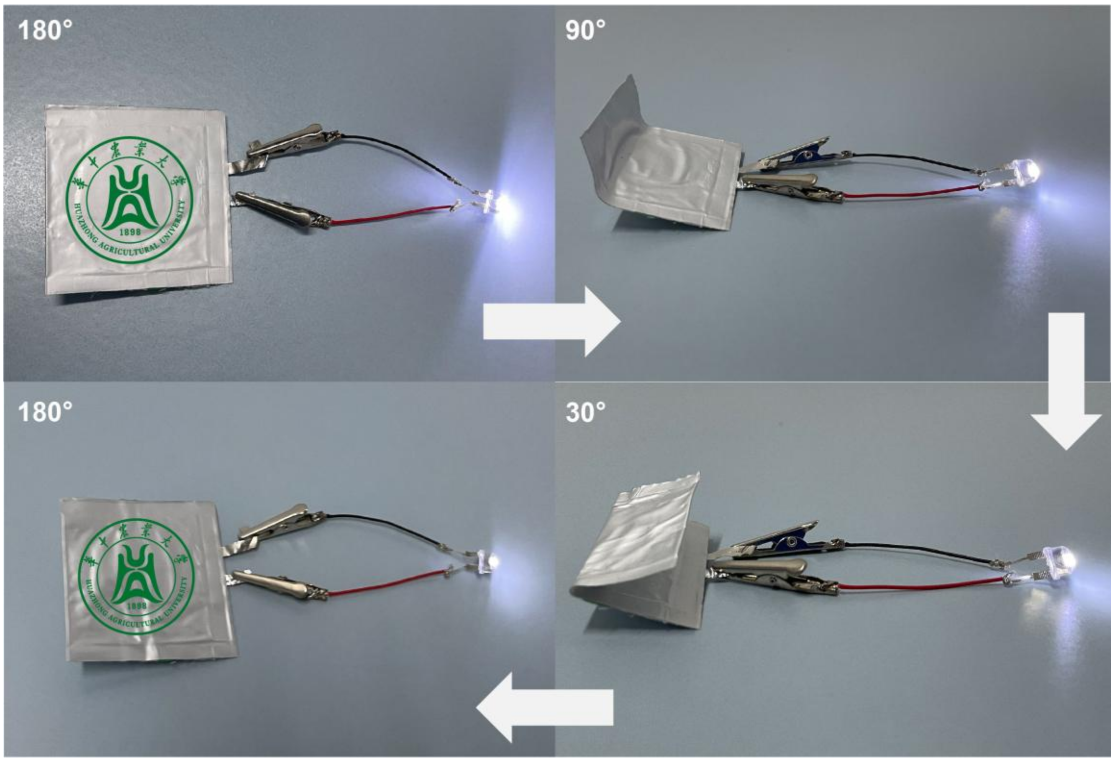


**Figure S35.** Bending test of carbon aerogel/Li|LFP pouch cell.

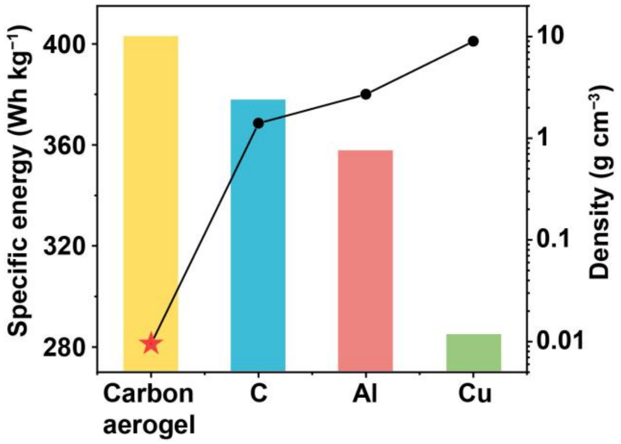


**Figure S36.** Gravimetric energy density and density comparison of the cells with different current collectors. The energy density is estimated based on Li-$LiFePO_4$ (LFP) batteries. The calculation method can be referred to **Table S1**.

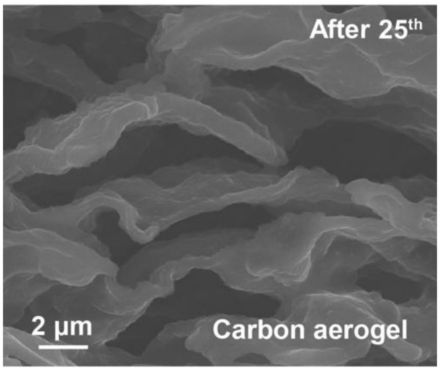


**Figure S37.** Cross-sectional image of the carbon aerogel/Li electrode of carbon aerogel/Li|SPAN full cell after 25 cycles at 0.2 C.

**Table S1.** Detailed analysis on the obtained EIS spectra of carbon aerogel and MXene-based Li|Li symmetric cells before cycle and after 25 cycling.

| Electrodes | Before cycle | | After 25 cycling | |
|---|---|---|---|---|
| | $R_{SEI}$ (Ω) | $R_{ct}$ (Ω) | $R_{SEI}$ (Ω) | $R_{ct}$ (Ω) |
| Carbon aerogel | 28.1 | 5.0 | 9.0 | 3.5 |
| MXene | 36.6 | 14.4 | 26.1 | 10.11 |

**Table S2.** The LFP full cell cycle life of various host materials.

| Electrode | Current density | Cycle number (n) | Capacity retention (%) | Ref. |
|---|---|---|---|---|
| **Carbon aerogel** | **1 C** | **300** | **90%** | **This work** |
| Graphite anode | 1 C | 300 | 59% | [9] |
| Zn-NC@MXene | 1 C | 100 | 87% | [10] |
| MXene/liquid metal film | 1 C | 200 | 78% | [11] |
| Por-PN-COF-Li | 1 C | 200 | 74% | [12] |
| MLFLi | 1 C | 200 | 78% | [13] |
| Li@$Ti_3C_2T_x$/g-$C_3N_4$ | 1 C | 150 | 84% | [14] |
| NOMC-Ni | 1 C | 350 | 83% | [15] |
| ZOS-CF | 1 C | 400 | 87% | [16] |
| Li-NCH@CF | 1 C | 150 | 90% | [17] |

**Table S3.** Detailed analysis on the obtained EIS spectra of LFP full cells for carbon aerogel and MXene electrodes before cycle and after 25 cycling.

| Electrodes | Before cycle<br>$R_{SEI}$ (Ω) | After 25 cycling<br>$R_{SEI}$ (Ω) |
|---|---|---|
| Carbon aerogel | 34.8 | 31.3 |
| MXene | 53.6 | 44.2 |

**Table S4.** Density of different current hosts.

| Hosts | Density (g $cm^{-3}$) |
|---|---|
| Carbon aerogel | 0.0095 |
| C | 1.4 |
| Al | 2.7 |
| Cu | 8.96 |

The gravimetric energy density of the full cells shown in Figure S23 is calculated according to previous article. Pouch cells with 20-stacked layers of cathode are used as the calculation model. The surface area of the pouch cell is 70 mm×41.5 mm. The capacity of the LFP cathode is 2.98 Ah, corresponding to a single layer areal capacity of 5 mA h $cm^{-2}$. The single layer masses of Li foil, Cu foil, Al foil, PP separator, and LFP used for assembling the pouch cell are 0.031, 0.156, 0.094, 0.029, and 1.068 g, respectively. Therefore, the total masses of Li foil, Cu foil, Al foil, PP separator, and LFP in the pouch cell model are 0.031×20, 0.156×10, 0.094×11, 0.029×20, 1.068×20 g.

The masses of the 20 μm carbon aerogel, C, Al, and Cu current collectors are 0.0005, 0.081, 0.16, and 0.52 g, respectively. Therefore, the total masses of carbon aerogel, C, Al, and Cu current collectors in the pouch cell model are 0.01, 1.63, 3.14, and 10.41 g, respectively.

The gravimetric energy density (W) of the pouch cell can be calculated as $W=E_{cell}C_{cell}/m_{cell} = (3.4\ V\times 2.98\ Ah)/[(25.15+n)\times 10^{-3}]\ (Wh\ kg^{-1})$.

(n= 0.01, 1.63, 3.14, and 10.41)

(Notes: $E_{cell}$ is the cell voltage, $C_{cell}$ is the cell capacity, and $m_{cell}$ is the total cell mass.)

**Table S5.** Parameters of carbon aerogel/Li|LFP pouch cell.

| Cell component | Specification | Parameters |
|---|---|---|
| Cathode | Active material | LFP |

| | | |
|---|---|---|
| | Active material mass loading (mg cm$^{-2}$) | 8 |
| | Active material ratio (wt %) | 80 |
| | Area capacity (mAh cm$^{-2}$) | 1.2 |
| | Dimension (2cm×3cm) | 6 |
| | Number of Layers | 1 |
| Separator | Total weight (mg) | 2.9 |
| Electrolyte | Total weight (mg) | 200 |
| Anode | Active material | carbon aerogel |
| | Area capacity (mAh cm$^{-2}$) | 6 |
| | Number of Layers | 1 |
| Pouch Cell | Discharge capacity (mAh) | 7.4 |
| | Mid-value voltage (V) | 3.4 |
| | Total weight (mg) | 347 |

The total weight excludes the package and tabs.

## References


[1] G. Kresse, *Phys. Rev. B* **1999**, *59*, 1758–1775.

[2] P. E. Blöchl, *Phys. Rev., B Condens Matter.* **1994**, *50*, 17953–17979.

[3] J. P. Perdew, K. Burke, M. Ernzerhof, *Phys. Rev. Lett.* **1996**, *77*, 3865–3868.

[4] J. P. Perdew, M. Ernzerhof, K. Burke, *J. Chem, Phys.* **1996**, *105*.

[5] S. Grimme, J. Antony, S. Ehrlich, H. Krieg, *J. Chem. Phys.* **2010**, *132*, 154104.

[6] H. Li, Y. Wu, C. Li, Y. Gong, L. Niu, X. Liu, Q. Jiang, C. Sun, S. Xu, *Appl. Catal. B* **2019**, *251*, 305–312.

[7] X. Huang, H. Yang, W. Liang, M. Raju, M. Terrones, V. H. Crespi, A. C. T. van Duin, S. Zhang, *Applied Physics Letters*. **2013**, *103*, 153901.

[8] H. Yang, X. Huang, W. Liang, A. C. T. van Duin, M. Raju, S. Zhang, *Chemical Physics Letters.* **2013**, *563*, 58–62.

[9] Yao Y X, Wan J, Liang N Y, et al. Nucleation and Growth Mode of Solid Electrolyte Interphase in Li-ion batteries. J. Am. Chem. Soc. 2023, 145, 14, 8001.

[10] He Q, Li Z, Wu M, et al. Ultra-uniform and functionalized nano-ion divider for regulating ion distribution toward dendrite-free lithium-metal batteries. Advanced Materials. 2023, 35, 39, 2302418.

[11] Wei C, Fei H, Tian Y, et al. Isotropic Li nucleation and growth achieved by an amorphous liquid metal nucleation seed on MXene framework for dendrite-free Li metal anode. Energy Stor. Mater. 2020, 26, 223.

[12] Zheng S, Fu Y, Bi S, et al. Three-Dimensional Covalent Organic Framework with Dense Lithiophilic Sites as Protective Layer to Enable High-Performance Lithium Metal Battery. Angewandte Chemie International Edition. 2025, 64, e202417973.

[13] Wei C, Fei H, Tian Y, et al. Isotropic Li nucleation and growth achieved by an amorphous liquid metal nucleation seed on MXene framework for dendrite-free Li metal anode. Energy Storage Materials. 2020, 26, 223-233.

[14] F. Zhao, P. Zhai, Y. Wei, et al. Constructing Artificial SEI Layer on Lithiophilic MXene Surface for High-Performance Lithium Metal Anodes. Adv. Sci. 2022, 9, 2103930.

[15] Huang W, Liu S, Yu R, et al. Single-Atom Lithiophilic Sites Confined within Ordered Porous Carbon for Ultrastable Lithium Metal Anodes. Energy & Environmental Materials, 2023, 6, e12466.

[16] Liu X, Tan H, Li Y, et al. Constructing Fast Ion/Electron Conducting Pathway within 3D Stable Scaffold for Dendrite-Free Lithium Metal Anode. Advanced Functional Materials, 2025, 35, 2420382.

[17] Chen C, Guan J, Li N W, et al. Lotus-root-like carbon fibers embedded with Ni–Co nanoparticles for dendrite-free lithium metal anodes. Advanced Materials, 2021, 33, 2100608.
